# Ground Moving Target Imaging via SDAP-ISAR Processing: Review and New Trends

**DOI:** 10.3390/s21072391

**Published:** 2021-03-30

**Authors:** Marco Martorella, Samuele Gelli, Alessio Bacci

**Affiliations:** 1 Department of Information Engineering, University of Pisa, 56122 Pisa, Italy; 2 Radar and Surveillance Systems National Laboratoy, CNIT, 56122 Pisa, Italy; sgelli@cnit.it; 3 Leonardo S.p.A., 50013 Firenze, Italy; alessio.bacci.85@gmail.com

**Keywords:** GMTI, radar, radar imaging, STAP, SAR, ISAR

## Abstract

Ground moving target imaging finds its main applications in both military and homeland security applications, with examples in operations of intelligence, surveillance and reconnaissance (ISR) as well as border surveillance. When such an operation is performed from the air looking down towards the ground, the clutter return may be comparable or even stronger than the target’s, making the latter hard to be detected and imaged. In order to solve this problem, multichannel radar systems are used that are able to remove the ground clutter and effectively detect and image moving targets. In this feature paper, the latest findings in the area of Ground Moving Target Imaging are revisited that see the joint application of Space-Time Adaptive Processing and Inverse Synthetic Aperture Radar Imaging. The theoretical aspects analysed in this paper are supported by practical evidence and followed by application-oriented discussions.

## 1. Introduction

Synthetic Aperture Radar (SAR) exploits the radar platform motion to form a large antenna aperture and, therefore, to provide high resolution images of an illuminated scene [1]. SAR systems have been widely used for various Earth observation applications, including geoscience, disaster monitoring, homeland security as well as in military contexts. More specifically, in homeland security and military-related scenarios, the attention is often paid to moving human-made targets, often addressed as non-cooperative targets. Similarly to the case of a photographic camera, moving targets typically appear defocused in SAR images. This is mainly due to the fact that a standard SAR processor is not designed to account for target’s motions. A solution to the imaging of moving targets is proposed in [2] in which Inverse SAR (ISAR) processing is successfully applied to targets detected within SAR images. Such solution, though, only considered maritime targets, which are much easier to detect than ground targets because of the reduced clutter intensity of the former with respect to the latter. In fact, in the presence of ground clutter and, particularly when considering slow moving targets, the echo of the latter overlaps with that of the ground clutter, which is typically much stronger. Another approach for SAR ground moving target imaging with inverse SAR scenario is suggested in [3] where a generalised inverse synthetic aperture radar (GISAR) geometry is addressed. Well-established methods for separating moving targets from stationary clutter in single-channel SAR systems are based on a Doppler analysis. More specifically, the signal relative to a moving target and that coming from the stationary clutter may be separated based on their spectral occupancy [4,5]. Such techniques are based on the assumption that the radar Pulse Repetition Frequency (PRF) is high enough to obtain a region in the Doppler frequency domain that is free of the static scene components. Doppler-based techniques can be readily applied to single-channel SAR data although they do not prove very effective. First of all, for these techniques to be applicable, high PRFs must be transmitted. Unfortunately, high PRFs may significantly reduce the SAR swath as well as they may increase the amount of data to be processed. Moreover, such techniques fail when attempting to detect slow moving targets. In fact, slow-moving targets generate low Doppler frequencies, which fall completely within the Doppler bandwidth of the stationary clutter, therefore not producing the required spectral separation. Other ground moving target detection techniques that are readily applicable to single-channel SAR systems are based on change detection. Such techniques make use of different looks of the same scene at different times [6]. Although they may be very effective, their implementation requires two passages over the same zone at different times, which complicates the overall acquisition mission. Moreover, an effective change detection needs a fine image co-registration, which is not always simple and easy. Last but not least, this method only leads to a detection that cannot be related to a real-time event but can only confirm that a target has moved in or out of a certain position in the time between the two passages. Additional ideas are proposed in [7], where the motion of moving targets is exploited for improving resolution and enhance their detectability, and in [8] where the motion of controllable illumination is exploited to obtain high-resolution imaging through a small effective aperture and therefore enhance signal to clutter ratio. When spatial Degrees of Freedom (DoFs) are available, such as in the case of multi-channel radar systems, more powerful techniques can be devised. These techniques exploit the ability to collect multiple spatial samples of the target’s echoes. This can be obtained by means of radar systems that employ multiple antenna and receiving channel elements. Multiple spatial samples are then mixed with multiple time samples, i.e., echoes collected at different Pulse Repetition Interval (PRIs), and processed jointly to reduce or even suppress strong ground clutter components. Displaced Phase Centre Array (DPCA) [9,10], Along Track Interferometry (ATI) [11,12,13], Space Time Adaptive processing (STAP) and Time-Frequency Transforms (TFT) [14,15,16] are examples of multichannel SAR techniques for mitigating the effects of stationary clutter. Particularly, STAP techniques have proven to be very effective in terms of their ability to suppress stationary clutter and have been widely used to detect slowly moving ground targets [17,18,19].

In the more recent years, with the development of multichannel M-SAR (SAR) systems, applications of Space Time Adaptive Processing to imaging systems have attracted the attention of many radar scientists and engineers. The authors of [20,21] derived an optimum space-time processing for moving target detection in SAR images and compared it against a number of reduced rank methods. Pre- and post-Doppler STAP were introduced by Rosenberg for joint jammer and clutter cancellation in multi-channel SAR images [22,23,24]. As a result of extensive studies and assessments, STAP and all its derived approaches are to be considered the most effective techniques for the detection of slow-moving ground targets.

Much attention has also been paid to the problem of clutter heterogeneity and limited availability of training data. Both these factors can drastically reduce the clutter-rejection performance of STAP. In recent years, several techniques have been developed to solve the problem of the lack of training data for an effective estimation of the clutter covariance matrix. In [25], a priori knowledge is exploited to effectively estimate the clutter covariance matrix, whereas, in [26], a method based on a small number of secondary samples is proposed. Differently, a method for the exploitation of additional training data has been proposed in [27], where additional data is obtained by means of a diverse waveform pulse compression. On the other hand, in a heterogeneous clutter environment, the clutter statistics are range-dependent and, therefore, the selected training data may have a different characteristic with respect to that of the area under test. Improper training data selection and the presence of non-stationary interference have been addressed, respectively, in [28] and [29], where a post-Doppler parametric adaptive matched filter and STAP based on piecewise sub-apertures have been proposed as solutions. Clutter range dependence, which involves a strong heterogeneity in the training data, is also present in forward-looking airborne SAR. In [30], an adaptive Doppler compensation to mitigate the degraded STAP performance has been proposed.

Only very recently, STAP has been investigated as a means to form images of ground moving targets. A combination of STAP and ISAR techniques has been proposed in [31] to obtain well-focussed images of moving targets when using a multi-channel SAR system. The approach in [31] has been formulated in the classic space/slow-time domain although, as introduced subsequently, a more interesting and effective implementation can be obtained in the space Doppler domain, [32,33].

This review paper collects a number of concepts and results that provide the author’s view and solution to the problem of imaging moving targets against strong clutter. Emphasis is given to both the theoretical aspects and practical implementation with real data-based case studies as evidence of the validity of the proposed concepts, architectures and algorithms. The paper is organised in order to illustrate theoretical findings through signal modelling and processing and to provide evidence of results based on real data collected in a number of airborne radar scenarios. In more detail, Section 2 provides the mathematical background that is necessary to fully understand the system concepts and architectures as well as the derived signal processing techniques. Subsequently, Section 3 addresses and validates the SDAP-ISAR technique on a set of real data acquired with a multi-channel SAR system. Section 4 illustrates the Virtual SDAP approach, which allows to apply, under certain conditions, SDAP by using only a single channel radar system. Virtual SDAP is also validated by using real data in Section 4. Section 5 introduces the concept of Cognitive SDAP through the definition of a cognitive multi-channel radar architecture and presents some evidence of its validity by using real data. Conclusions are finally drawn in Section 6.

## 2. Background of Ground Moving Target Imaging

This section provides some background knowledge that is fundamental for introducing and comprehending the concepts that will follow in this paper. In particular, it focuses on multichannel and non-cooperative target imaging techniques and on the definition of signal and clutter models that will be used throughout the rest of this paper. More specifically, a formulation of a multi-static version of the range-Doppler image formation algorithm will be provided, followed by a review of an effective imaging technique to produce high resolution images of moving targets.

### 2.1. Multichannel ISAR Signal Model

The multichannel ISAR signal model, addressed briefly in this section, is a generalisation of the model introduced in [34] where a configuration with two orthogonal baseline for 3D target reflectivity function reconstruction was considered.

**Figure 1:**
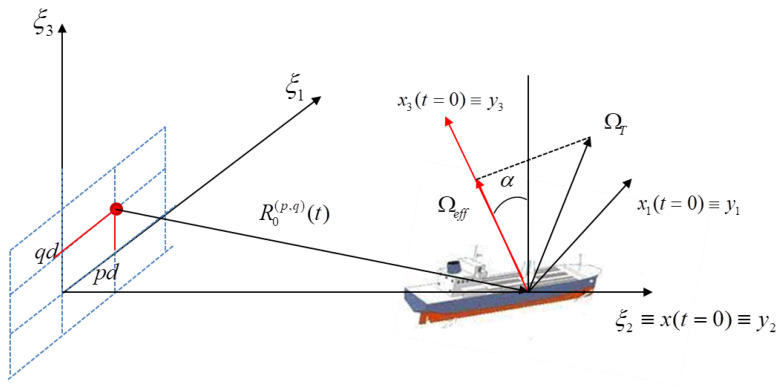
Multichannel ISAR geometry.

Figure 1 shows a geometry where a bidimensional array carrying by a moving platform observe a scene in which a non-cooperative moving target is present. The moving platform can be either an airborne or a spaceborne platform. In this geometry three different reference systems can be easily defined. The reference system $T_{\xi }$ has its origin in the phase centre of the transmitter and the $\xi_{2}$ axis parallel to the radar Line of Sight (*LoS*). Moreover the $\xi_{1}$ and the $\xi_{3}$ axes correspond, respectively, to the horizontal and vertical baselines. As detailed in [35], the target’s own motion can be modelled as a superimposition of a translational motion component, namely $R_{0}\left(t\right)$, and rotational motion velocity vector, namely $\Omega_{T}\left(t\right)$. Both components are considered to be applied to the same reference point of the target. The projection of $\Omega_{T}\left(t\right)$ on the plane orthogonal to the LoS is namely the effective rotation vector $\Omega_{eff}\left(t\right)$ and represents the aspect angle variation that can be observed by the radar. The other reference system, $T_{x}$, which appears in Figure 1, is centred in the target’s reference point and has the $x_{2}$ axis directed along the radar LoS and the $x_{3}$ axis oriented along the direction of $\Omega_{eff}\left(t\right)$. The angle between the axes $\xi_{3}$ and $x_{3}$ is the angle α. It is worth pointing out that $x_{3}$ is chosen to complete the orthogonal Cartesian triad. Finally, the reference system $T_{y}$ is fixed with the target and it is defined so that it coincides with $T_{x}$ at t=0 and rotates with respect to $T_{x}$ depending on the relative LoS direction. In this case, all the antenna elements act as transmitting and receiving antennas. However, as demonstrated in [36], if only one antenna acts as transmitter and all the other antenna elements are receive-only elements, it is still possible to define an equivalent monostatic configuration for each transmitter-receiver bistatic couple. The inter-element spacing *d* between antenna elements is considered to be the same in both dimensions. The couple (p,q) denotes the element position, i.e., $\xi_{1}=pd$, $\xi_{2}=qd$ where the indexes $p=-\frac{P}{2},...,\frac{P}{2}-1$ and $q=-\frac{Q}{2},...,\frac{Q}{2}-1$ define the element position within the array. If a monostatic (or equivalent monostatic) configuration is considered and by assuming that the straight iso-range approximation is verified (this is always true in far field [35]), the phase of the received signal from a single transmitter/received positioned at the centre of the reference system $T_{\xi }$ can be written as follows:

(1) $$\phi \left(y,t,f\right)=\frac{4\pi f}{c}\left(R_{0}\left(t\right)+y\cdot i_{LoS}\left(t\right)\right)$$

where $R_{0}\left(t\right)$ is the relative distance between the moving platform and the target reference point at a generic time *t*, y represent the position of the scatterer in the $T_{y}$ reference system and $i_{LoS}\left(t\right)$ is the unit vector along the radar LoS at time *t*. Consequently, the signal received by the array element (p,q) can be express as follows:

(2) $$S_{R}^{\left(p,q\right)}\left(f,t\right)=W\left(f,t\right)\int_{V}\sigma {\left(y\right)}^{\left(p,q\right)}e^{-j\frac{4\pi f}{c}\left[R_{0}^{\left(p,q\right)}\left(t\right)+y\cdot i_{LoS_{\xi }}^{\left(p,q\right)}\left(t\right)\right]}dy$$

where (f,t) represent the range frequency and the slow-time respectively. A rotation matrix $M_{\xi x}$ is here introduced to generate a rotation of $T_{x}$ with respect to $T_{\xi }$ of an angle α, and can be written as:

(3) $$M_{\xi x}=\left[\begin{array}{ccc} \cos\left(\alpha \right) & 0 & \sin\left(\alpha \right)\\ 0 & 1 & 0\\ -\sin\left(\alpha \right) & 0 & \cos\left(\alpha \right) \end{array}\right]$$

It is possible to obtain the LoS unit vector $i_{LoS_{x}}^{\left(p,q\right)}\;\left(t\right)$ in the reference system $T_{x}$ as the normalised difference between the positions of each sensor and the origin of $T_{x}$ by means of the rotation matrix $M_{\xi x}$:

(4) $$i_{LoS_{x}}^{\left(p,q\right)}\left(t\right)=M_{\xi x}i_{LoS_{\xi }}^{\left(p,q\right)}\left(t\right)=\frac{1}{C}\left[\begin{array}{c} -pdcos\left(\alpha \right)-qdsin\left(\alpha \right)\\ R_{0}\left(t\right)\\ pdsin\left(\alpha \right)-qdcos\left(\alpha \right) \end{array}\right]$$

where

(5) $$C=\sqrt{R_{0}^{2}\left(t\right)+{\left(pd\right)}^{2}+{\left(qd\right)}^{2}}\approx R_{0}\left(t\right)$$

is the normalisation factor. By considering that the radar-target distance is much larger than the array size, it is possible to approximate the normalisation factor *C* as in the right side of Equation (5). Moreover, for small observation times, $R_{0}\left(t\right)\approx R_{0}\left(0\right)=R_{0}$. As detailed in [35], the scatter position x(t) can be expressed as follows:

(6) x(t)≅a+b+ct=y+ct

where $a=\frac{\left(\Omega_{T}\cdot y\right)}{\Omega_{T}^{2}}\Omega_{T}$, $b=y-\frac{\left(\Omega_{T}\cdot y\right)}{\Omega_{T}^{2}}\Omega_{T}$, $c=\Omega_{T}\times y$.

By considering Equation (3), the inner product can be rewritten as:

(7) $$y\cdot i_{LoS_{\xi }}^{\left(p,q\right)}\left(t\right)=x\left(t\right)\cdot i_{LoS_{x}}^{\left(p,q\right)}\left(t\right)=K_{0}^{\left(p,q\right)}+K_{1}^{\left(p,q\right)}t$$

where

(8) $$\begin{array}{c} K_{0}^{\left(p,q\right)}=y_{2}-\frac{d}{R_{0}}\left[y_{1}\left(pcos\left(\alpha \right)+qsin\left(\alpha \right)\right)+y_{3}\left(qcos\left(\alpha \right)-psin\left(\alpha \right)\right)\right]\\ K_{1}^{\left(p,q\right)}=c_{2}-\frac{d}{R_{0}}\left[c_{1}\left(pcos\left(\alpha \right)+qsin\left(\alpha \right)\right)+c_{3}\left(qcos\left(\alpha \right)-psin\left(\alpha \right)\right)\right] \end{array}$$

and where $c_{2}=\Omega_{eff}y_{1}$ [33,37].

The ISAR point spread function (PSF) related to a single scatter $y^{\left(k\right)}$ at the generic receiving channel (p,q) is obtained through a two-dimensional inverse Fourier transform (2D-IFT) of the signal after motion compensation, and can be expressed as follows:

(9) $$\begin{array}{cc} I^{\left(p,q\right)}\left(\tau ,\nu \right) & =B\cdot T_{obs}\cdot \sigma \left(y_{1}^{\left(k\right)},y_{2}^{\left(k\right)}\right)\cdot e^{j2\pi f_{0}\left(\tau -\frac{2}{c}K_{0}^{\left(p,q\right)}\right)}\times \\ \; & \mathrm{sinc}\left[T_{obs}\left(\nu +\frac{2f_{0}}{c}K_{1}^{\left(p,q\right)}\right)\right]\cdot \mathrm{sinc}\left[B\left(\tau -\frac{2}{c}K_{0}^{\left(p,q\right)}\right)\right] \end{array}$$

It should be noted that, when the array size is much smaller than the radar-target distance, $K_{0}$ and $K_{1}$ can be approximated as:

(10) $$\begin{array}{c} K_{0}^{\left(p,q\right)}=y_{2}\\ K_{1}^{\left(p,q\right)}=c_{2}=\Omega_{eff}y_{1} \end{array}$$

The model presented here can be simplified in the case of linear arrays, which can be derived from the general case by posing q=0.

An integrated image can be obtained by summing up the resulting images at the output of each of the *P* channels. The sum can be performed effectively only if all the *P* channels are phase-aligned. Theoretically, this can only be true for a single point on the ground. In practice a tolerance in the phase error can be introduced that allows of a region on the ground to be effectively imaged with a linear array. Such a bound poses a constraint directly on the array size. As a consequence, the maximum array size can be found by imposing the maximum tolerable phase difference among the images:

(11) $$\frac{4\pi }{\lambda }\left(P-1\right)\frac{d}{R_{0}}\left(y_{1}\cos\alpha -y_{3}\sin\alpha \right)\leq \frac{\pi }{8}$$

which leads to:

(12) $$D_{array}\leq \frac{\lambda R_{0}}{32\left(y_{1}\cos\alpha -y_{3}\sin\alpha \right)}$$

where $\lambda =\frac{c}{f_{0}}$ and where $\left(y_{1}\cos\alpha -y_{3}\sin\alpha \right)$ represent the target size in the $\xi_{1}$ dimension. In the event that the target size along the $\xi_{1}$ dimension does not satisfy the bound expressed in Equation (12), it is still possible to apply the described method by splitting the entire illuminated area into regions with a smaller size such as to satisfy Equation (12). Then, for each of these regions, a separate image focus point should be used as a reference point. Distortions appear in the image, in case the constraint is not met. A closed-form solution of the attenuation term can be calculated as follows:

(13) $$\begin{array}{cc} I(y_{1},y_{2}) & =\sum_{p=-\frac{P}{2}}^{\frac{P}{2}-1}I^{\left(p\right)}\left(y_{1},y_{2}\right)\\ \; & =I^{\left(0\right)}\left(y_{1},y_{2}\right)\sum_{p=-\frac{P}{2}}^{\frac{P}{2}-1}e^{j\frac{4\pi }{\lambda }\frac{d}{R_{0}}y_{1}p}\\ = & I^{\left(0\right)}\left(y_{1},y_{2}\right)e^{+j\frac{4\pi }{\lambda }\frac{dPy_{1}}{2R_{0}}}\sum_{p=0}^{P-1}e^{j\frac{4\pi }{\lambda }\frac{d}{R_{0}}y_{1}p} \end{array}$$

In order to simplify the notation and to make it clearer for the reader, we will show below the case with α=0. After some mathematical manipulations, Equation (13) can be expressed as follows

(14) $$I\left(y_{1},y_{2}\right)=I^{\left(0\right)}\left(y_{1},y_{2}\right)e^{j\frac{2\pi Pd}{\lambda 2R_{0}}y_{1}}\frac{\sin\left(\frac{2\pi dy_{1}P}{\lambda R_{0}}\right)}{\sin\left(\frac{2\pi dy_{1}}{\lambda R_{0}}\right)}$$

**Figure 2:**
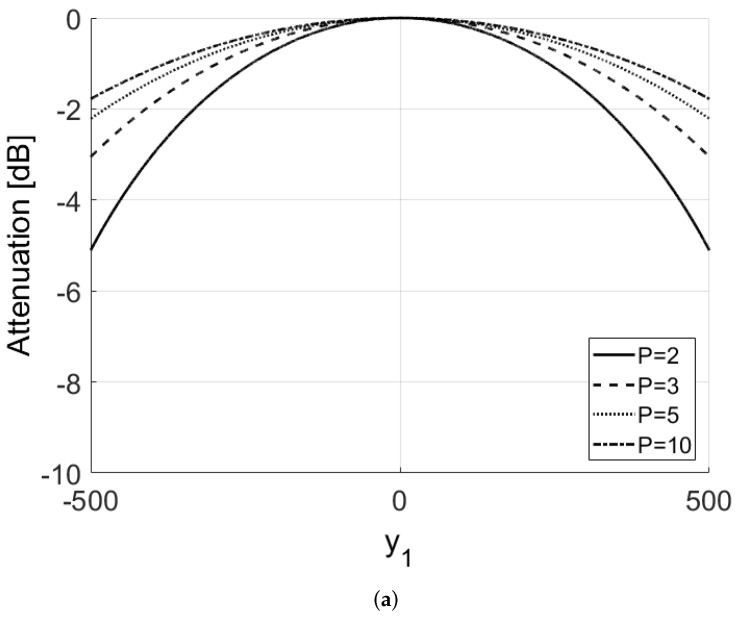

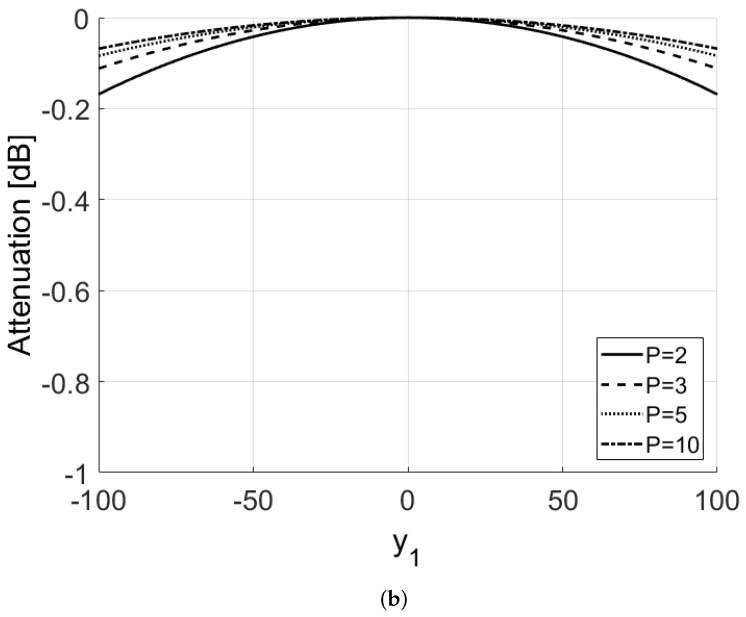
Attenuation factor. (**a**) The attenuation term *J*(*y*_1_) is shown for the radar center-scene distance, *R*_0_ = 5 km, a carrier frequency, *f*_0_ = 10 GHz. (**b**) Represent a zoom-in version of subplot(a) Reproduced with permission from Alessio Bacci, *Optimal Space Time Adaptive Processing for Multichannel Inverse Synthetic Aperture Radar Imaging*, PhD Thesis; published by University of Pisa and University of Adelaide, Australia 2014.

As explained above, the term $J\left(y_{1}\right)=\frac{\sin\left(\frac{2\pi dy_{1}P}{\lambda R_{0}}\right)}{\sin\left(\frac{2\pi dy_{1}}{\lambda R_{0}}\right)}$ produces a distortion in the image amplitude due to the phase misalignment. The attenuation term $J(y_{1})$ is shown in Figure 2 for a distance equal to $R_{0}=5$ km and a carrier frequency $f_{0}=10$ GHz. The inter-element distance is instead obtained by imposing the condition expressed in Equation (12), with $y_{1}=100$ m, which yields:

(15) $$d=\frac{\lambda R_{0}}{32y_{1}\left(P-1\right)}$$

Equation (12) produce a loss of 0.2 dB within a distance of 100 m from the focusing point, indicating that this condition may be quite restrictive if longer synthetic apertures are used.

### 2.2. High Resolution Imaging of Non-Cooperative Moving Targets

**Figure 3:**
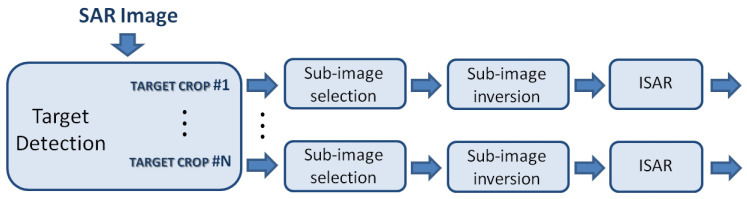
Processing chain of detection and refocusing processor.

Standard SAR processing implies the assumption of a known platform trajectory and a static scenario during the synthetic aperture formation. Under these assumptions, a direct motion compensation can be applied. This produces a highly focused image of the observed static area by means of coherent integration of the received signal. On the other hand, a non-cooperative moving target would not appear well-focussed and it would be displaced in the SAR image because the relative motion between the moving platform and the moving target is not compensated [35,38]. Many techniques [39,40,41,42] have been proposed in the literature to overcome the problem of the phase compensation between the radar moving platform and a non-cooperative target. Some of these are based on restrictive assumptions, which constrain the target to move along rectilinear trajectories, whereas others require the existence of multiple prominent scatterers on the target. ISAR processing can be a viable solution to the problem of focusing moving targets that are present in a SAR scene. Differently from the SAR case, where fine cross-range resolution is obtained by using the platform motion during the Coherent Processing Interval (CPI), in the ISAR case, the radar is assumed fixed to the ground and the cross-range resolution is obtained by exploiting the movement of the target [38]. It is worth pointing out that the relative motion between radar and target is estimated and compensated by the ISAR processing during the image formation process and no a priori information about radar-target relative motion is required. For this reason, a method has been proposed in recent years that exploits the ISAR technique to refocus moving targets in SAR images for both monostatic and bistatic configuration [2,43]. A processing block scheme is depicted in Figure 3 that describes the signal processing steps that are needed to refocus a blurred image of a non-cooperative moving target. The required signal processing is composed of the main steps described follows:

- **Target Detection**
  The target, independently of how well is focussed, must be detected first. Differently from maritime scenario, where the backscatter of sea clutter is typically weaker than the target’s return, the detection of moving target in ground clutter scenarios can be critical since ground clutter can often mask the target completely.
- **Sub-Image Selection**
  After the first step (of target detection), each detected target must be extracted from the SAR image. This is done by separating the target’s return from clutter and other target’s returns. This is a fundamental step since each target has its own motion, which is different from that of the other targets and, therefore, its signal must be processed independently of the others. A number of sub-images equal to the number of detected targets can be obtained by processing each target’s return in parallel with separate instances of the ISAR processor.
- **Sub-Image Inversion**
  A conversion from the image domain to the raw data domain is required as already implemented ISAR processors accept raw data as input. Depending on the algorithm used to form the SAR image, different algorithms can be used for image inversion.
  The following conditions will be here assumed: (1) the straight iso-range (or far field) approximation holds true and (2) the total aspect angle variation can be considered small enough and then the effective rotation vector can be considered constant during the CPI. Generally the received signal is defined on a polar grid in the Fourier domain. However under these approximations the Fourier domain can be approximated with a rectangular and regularly-sampled grid. Consequently, the two-dimensional Fast Fourier Transform (2D-FFT) can be used to reconstruct the image through the range Doppler algorithm. In this case the Inverse range-Doppler (IRD), which consist of a two-dimensional inverse Fourier transform, is the most viable inversion algorithm and can be easily implemented by means of an inverse 2D-FFT.
  A number of more accurate image reconstruction algorithms have been proposed in many years of SAR image formation research. A non-exhaustive but significant list of such algorithms follows: *Omega-k* also called *range migration algorithm* [1], *Range stacking* [44], *Time Domain Correlation* (TDC) [45] and Back-projection [1].
- **ISAR Processing**
  As mentioned about, after target detection, it is possible to separate the target contribution from both the contribution of clutter and that of other targets. Through the sub-image inversion step the raw data for each sub-image can be obtained. ISAR processing can be then applied to produce a high resolution image of the moving target. It is worth emphasizing that the SAR image formation processing focuses the static scene by compensating for the movement of the platform. Therefore, only the residual motion between the radar platform and the non-cooperative moving target needs to be compensated by means of ISAR processing.

#### ISAR Processing

**Figure 4:**
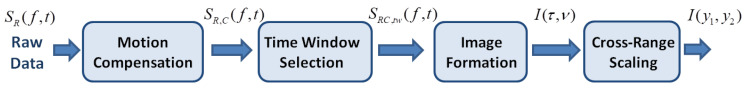
ISAR processing chain.

Figure 4 shows the main steps that compose the ISAR processor, which are briefly summarised below and detailed in the following paragraphs

- Motion Compensation;
- Time Window Selection;
- Image Formation;
- Cross-Range Scaling.

MotionCompensation

Different motion compensation techniques can be found in the literature. Some of them are summarised in [38]. The technique implemented here is the Image Contrast Based Autofocus (ICBA) algorithm, and aims estimating and compensating the target radial motion by maximising the Image Contrast (IC).

Briefly, the ICBA algorithm is a parametric autofocus technique where the problem of the target motion compensation, i.e., the estimation and the suppression of the term $R_{0}\left(t\right)$, is recast as an optimisation problem based on the Image Contrast (IC) maximisation. More details can be found in [46].

TimeWindowSelection

Under the assumption of a constant effective target rotation vector and small total aspect angle variation, the RD algorithm can be applied. However, in some cases, these approximations do not hold true. A viable solution to this problem is to take into account a temporal window in the slow time domain that can be used to select a suitable time interval. In fact, if the time interval is small enough, the RD processing can be applied effectively for the image formation. However, a large window is instead required in order to obtain a fine cross-range resolution.

In [47], a solution for the optimal selection of the length and the position of the window to obtain an image with highest focus (largest image contrast), is addressed. It should be mentioned that the IC allows for the largest time window to be selected for the finest resolution to be obtained before aspect angle variations start producing their negative effects in terms of image distortions.

ImageFormation

Given the previous processing steps, the RD algorithm, which is implemented through a two-dimensional inverse fast Fourier transform, is used for the image formation as follow:

(16) $$I\left(\tau ,\nu \right)=2D-IFT\left[S_{R,C}\left(f,t\right)\right]$$

where $S_{R,C}\left(f,t\right)$ is the received signal after motion compensation in which (f,t) represent the range frequency and the slow-time respectively, while $I\left(\tau ,\nu \right)$ represents the ISAR image and 2D−IFT represents the two-dimensional Inverse Fourier Transform.

CrossRangeScaling

Without any further refinement, an ISAR image is obtained in the time delay-Doppler domain, i.e., $I\left(\tau ,\nu \right)$, by appying the RD algorithm. Nevertheless, in order to determine some target’s geometrical feature, such as the size, a spatially scaled image should be presented, i.e., an image in the range and cross-range domain. Firstly, as shown in [38], the well-known relationship, $y_{2}=\frac{c\tau }{2}$, can be used to easily scale the image from the delay domain to the range domain. The cross-range scaling, instead, requires the knowledge of the target’s effective rotation vector magnitude, namely $\Omega_{eff}$, which is not known a priori and cannot be measured directly.

Under the assumption of a constant target rotation vector in the CPI, the chirp rate produced by the target scatterers can be related to the effective rotation vector. If a sufficient number of scatterers can be extracted from the ISAR image and, therefore, an equal number of chirp rates estimated, the modulus of the effective rotation vector can be estimated by applying a simple Least Square Error (LSE) estimator. In [48], an effective algorithm has been introduced that solves the cross-range scaling problem.

## 3. Ground Moving Target Imaging via Space-Doppler Adaptive Processing

As already mentioned, STAP allows for stationary clutter to be suppressed in order to detect ground moving targets. In this section, we will shift the focus to target imaging rather than target detection. For this reason, a new technique has been introduced by the authors in [33] where a different implementation of STAP has been developed and combined with ISAR processing to form well-focused images of non-cooperative moving targets, which will be recalled in this section. Firstly, a method will be implemented that will allow for ISAR processing to be applied to a clutter-mitigated SAR image. Then, a sub-optimal approach will be introduced for an effective estimation of the clutter space-time covariance matrix. Finally, a modified version of the classical Space Time Adaptive Processing (STAP) [17], will be detailed as a result of the derivation of the range-Doppler image formation algorithm. As this modified version is directly implemented in the Doppler domain, it has been renamed Space-Doppler Adaptive Processing (SDAP).

The SDAP theoretical formulation will be derived for both the optimum and sub-optimum case.

### 3.1. Optimum Processing

**Figure 5:**
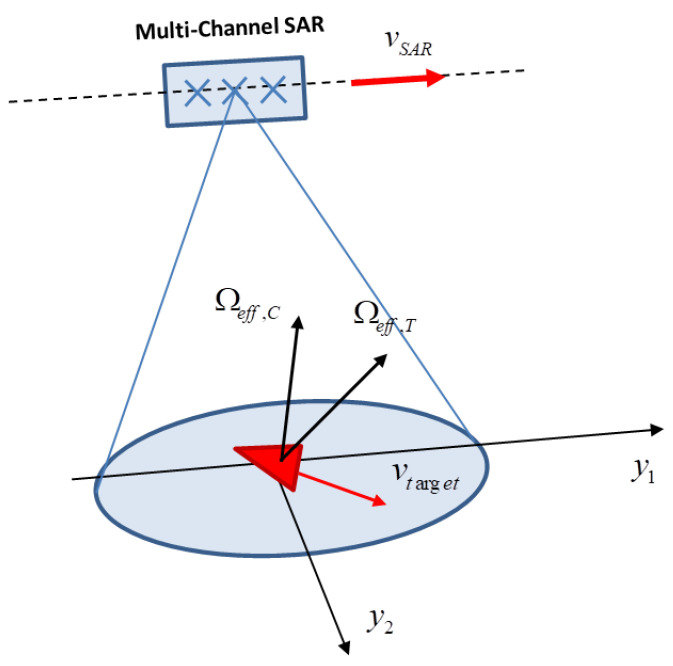
Acquisition geometry relative to a multichannel side-looking SAR system.

Figure 5 shows the acquisition geometry where a moving target is immersed in a stationeries clutter background. The signal received by the radar on the moving platform after Fourier transform (signal spectrum) can be expressed as follows:

(17) $$S\left(f,t\right)=S_{t}\left(f,t\right)+S_{c}\left(f,t\right)+N\left(f,t\right)$$

where $S_{t}\left(f,t\right)$ represents the target return, $S_{c}\left(f,t\right)$ is the clutter contribution and N(f,t) is an additive noise. $f\in \left[f_{0}-\frac{B}{2},f_{0}+\frac{B}{2}\right]$ and $t\in \left[\frac{-T_{obs}}{2},\frac{T_{obs}}{2}\right]$ denote the range frequency and the slow-time, respectively. It is worth pointing out that, according to Section 1, the multi-channel signal can be derived form the single channel signal model. The target return can be expressed as follows:

(18) $$S_{t}\left(f,t\right)=e^{-j\frac{4\pi }{\lambda }R_{0}\left(t\right)}\sum_{k=1}^{K}\sigma_{k}e^{-j\frac{4\pi }{\lambda }\left[K_{0,k}^{\left(p\right)}+K_{1,k}^{\left(p\right)}t\right]}$$

where both terms $K_{0}^{\left(p\right)}$ and $K_{1}^{\left(p\right)}$ are derived in Equation (8).

It is worth reminding that both $K_{0}^{\left(p\right)}$ and $K_{1}^{\left(p\right)}$ can be reasonably approximated as in Equation (10). In fact, in the case where the antenna dimension is smaller that the distance between the radar and the target, the LoS of each antenna element can be considered equivalent to the others.

Under this assumption the received signal relative to a moving target, namely $S_{t(f,t)}$, and the static background, namely $S_{c}\left(f,t\right)$, can be expressed as:

(19) $$S_{t}\left(f,t\right)=e^{-j\frac{4\pi }{\lambda }R_{0t}\left(t\right)}\sum_{k=1}^{K}\sigma_{k}e^{-j\frac{4\pi }{\lambda }\left[y_{2}^{\left(k\right)}+\Omega_{eff,t}y_{1}^{\left(k\right)}t\right]}$$

(20) $$S_{c}\left(f,t\right)=e^{-j\frac{4\pi }{\lambda }R_{0c}\left(t\right)}\underset{\left(y_{1},y_{2}\right)}{\;\int \int \;}\sigma \left(y_{1},y_{2}\right)e^{-j\frac{4\pi }{\lambda }\left[y_{2}+\Omega_{eff,c}y_{1}t\right]}dy_{1}dy_{2}$$

where the position of the reference point on the target, which includes both the motion of the target and the motion of the platform, is indicated with $R_{0t}\left(t\right)$, *k* indicates the index of a generic scatterer while the coordinates in cross-range and in range relatively to the $k^{th}$ scatterer are indicated, respectively, with $y_{1}^{\left(k\right)}$ and $y_{2}^{\left(k\right)}$. Finally, the platform and the target motions are included in the term $\Omega_{eff,t}$ which is the effective rotation vector.

It is worth highlighting that the proposed SDAP processing for clutter suppression and target imaging is based on the range- Doppler algorithm and that the straight-iso range approximation is mandatory to apply this processing. According to the theory, range resolution is related to the signal bandwidth. Then high range resolution can be obtained by exploiting wideband signals in transmission and by matched filtering the echoes. Moreover, through the RD processing, high azimuth resolution can be achieved [33,38]. Let consider a static scatter point placed in $\left(y_{1},y_{2}\right)$ and let $S_{t}\left(f,t\right)$ be the received signal. Then, the range-Doppler image formation can be obtained by means of a Fourier Transform, as above:

(21) $$u_{D}\left(f,\nu \right)=FT_{t}\left\{S_{t}\left(f,t\right)S_{ref}^{*}\left(f,t\right)\right\}$$

where $FT_{t}\{\}$ is the Fourier Transform along the slow time domain. Equation (21) can be also expressed via a convolution in the Doppler frequency domain:

(22) $$u_{D}\left(f,\nu \right)={\tilde{S}}_{ref}\left(f,-\nu \right)⊗{\tilde{S}}_{t}\left(f,\nu \right)$$

where

(23) $${\tilde{S}}_{t}\left(f,\nu \right)=FT_{t}\left\{S(f,t)\right\}$$

and

(24) $${\tilde{S}}_{ref}\left(f,\nu \right)=FT_{t}\left\{S_{ref}\left(f,t\right)\right\}$$

are the received signal and the reference signal after a Fourier transform, respectively.

Noticeably, Equation (22) shows that the image formation process via the range Doppler algorithm can be interpreted as a matched filtering in the Doppler frequency domain.

A discretised form $S\left(n,m\right)=S\left(n\Delta f,mT_{R}\right)$ can be used to express the proposed formulation. The indexes $n=\left[\frac{-N}{2},...,\frac{N}{2}-1\right]$ and $m=\left[\frac{-M}{2},...,\frac{M}{2}-1\right]$ represent the discrete frequency and the pulse index, respectively, whereas δf and $T_{R}$ represent the frequency sampling step and the Pulse Repetition Interval (PRI), in the same order. When considering a discretised domain, the RD processing can be rewritten as follows:

(25) $$u_{D}\left(n,m_{\nu }\right)=DFT_{m}\left\{S_{t}\left(n,m\right)S_{ref}^{*}\left(n,m\right)\right\}$$

where $m_{\nu }$ denotes the Doppler frequency index. Equivalently, the same can be written directly in the Doppler domain as a matched filtering operation, as follows:

(26) $$u_{D}\left(n,m_{\nu }\right)={\tilde{S}}_{t}\left(n,m_{\nu }\right){⊗}_{m_{\nu }}{\tilde{S}}_{ref}\left(n,-m_{\nu }\right)$$

where

(27) $${\tilde{S}}_{t}\left(n,m_{\nu }\right)=DFT_{m}\left\{S_{t}\left(n,m\right)\right\}$$

(28) $${\tilde{S}}_{ref}\left(n,m_{\nu }\right)=DFT_{m}\left\{S_{ref}\left(n,m\right)\right\}$$

In the last expression, $DFT_{m}$ represent the Discrete Fourier Transform operation along the discretised slow-time domain while the discrete convolution is denoted with ${⊗}_{m_{\nu }}$.

A vectorial form can be used to rewrite the matched filtering operation in Equation (26). After defining the signal vector, i.e., $\tilde{S}\left(n\right)$, and the reference vector, i.e., ${\tilde{G}}_{D}\left(n,m_{\nu }\right)$, as

(29) $$\tilde{S}\left(n\right)={\left[\tilde{S}\left(n,0\right),\tilde{S}\left(n,1\right),...,\tilde{S}\left(n,M-1\right)\right]}^{T}\in C^{M\times 1}$$

(30) $${\tilde{G}}_{D}\left(n,m_{\nu }\right)={\left[{\tilde{S}}_{ref}\left(n,m_{\nu }\right),...,{\tilde{S}}_{ref}\left(n,m_{\nu }-\left(M-1\right)\right)\right]}^{T}\in C^{M\times 1}$$

the vectorial form can be then obtained:

(31) $$u_{D}\left(n,m_{\nu }\right)={\tilde{G}}_{D}^{H}\left(n,m_{\nu }\right)\tilde{S}\left(n\right)$$

The achieved result can be extended in the case of a multichannel system. Consequently, the multichannel range-Doppler image formation can be expressed as follows:

(32) $$u_{D}\left(n,m_{\nu }\right)=\sum_{p=1}^{P}u_{D,p}\left(n,m_{\nu }\right)=\sum_{p=1}^{P}{\tilde{S}}_{t,p}\left(n,m_{\nu }\right){⊗}_{m_{\nu }}{\tilde{S}}_{ref,p}\left(n,-m_{\nu }\right)$$

Through a staking operation, first along the channel dimension, as expressed in Equations (33) and (34), and, then, along the Doppler frequency dimension, as shown in Equations (35) and (36), it is possible to express Equation (32) in a vectorial form as follows:

(33) $$\tilde{S}\left(n,m_{\nu }\right)=\frac{1}{P}{\left[{\tilde{S}}_{1}\left(n,m_{\nu }\right),{\tilde{S}}_{2}\left(n,m_{\nu }\right),...,{\tilde{S}}_{P}\left(n,m_{\nu }\right)\right]}^{T}\in C^{P\times 1}$$

(34) $${\tilde{S}}_{ref}\left(n,m_{\nu }\right)=\frac{1}{P}{\left[{\tilde{S}}_{ref,1}\left(n,m_{\nu }\right),{\tilde{S}}_{ref,2}\left(n,m_{\nu }\right),...,{\tilde{S}}_{ref,P}\left(n,m_{\nu }\right)\right]}^{T}\in C^{P\times 1}$$

(35) $$\tilde{S}\left(n\right)={\left[\tilde{S}\left(n,0\right),\tilde{S}\left(n,1\right),...,\tilde{S}\left(n,M-1\right)\right]}^{T}\in C^{MP\times 1}$$

(36) $${\tilde{G}}_{D}\left(n,m_{\nu }\right)={\left[{\tilde{S}}_{ref}\left(n,m_{\nu }\right),...,{\tilde{S}}_{ref}\left(n,m_{\nu }-\left(M-1\right)\right)\right]}^{T}\in C^{MP\times 1}$$

The Doppler matched filter can be then expressed as:

(37) $$u_{D}\left(n,m_{\nu }\right)={\tilde{G}}_{D}^{H}\left(n,m_{\nu }\right)\tilde{S}\left(n\right)$$

It is worth reminding that the straight iso-range approximation must be effective for this image formation processing to be effective. After applying the Doppler processing, in order to for a range-Doppler image, a last Fourier transform must be carried out along the range frequency dimension.

### 3.2. SDAP-ISAR

The application of optimum SDAP produces a weight vector that maximises the output SINR. Mathematically, the maximum SINR output can be then obtained by substituting the reference vector with the weight vector obtained through the application of SDAP

(38) $$u_{D}\left(n,m_{\nu }\right)={\tilde{W}}_{D}^{H}\left(n,m_{\nu }\right)\tilde{S}\left(n\right)$$

Practically, the optimum SDAP filter can be realised by means of the sample matrix inversion (SMI) implementation [49], as detailed in Equation (39):

(39) $${\tilde{W}}_{D}\left(n,m_{\nu }\right)=\gamma {\hat{R}}_{Dc}^{-1}{\tilde{G}}_{D}\left(n,m_{\nu }\right)$$

where the SINR at the filter output is not affected by the scalar parameter γ. Moreover, the estimation of the interference cross-power spectral matrix $R_{Dc}$, indicated with ${\hat{R}}_{Dc}$, can be obtained by exploiting $N_{r}$ target-free training data as follows:

(40) $${\hat{R}}_{Dc}=\frac{1}{N_{r}}\sum_{n_{r}=0}^{N_{r}-1}\tilde{Z}\left(n_{r}\right){\tilde{Z}}^{H}\left(n_{r}\right)\in C^{MP\times MP}$$

The vector $\tilde{Z}\left(n_{r}\right)$ represent the *n*th target-free range cell expressed in the Space-Doppler frequency domain.

In order to effectively implement SDAP to perform clutter suppression and high resolution imaging of moving targets, two considerations must be made and relative solutions identified. The first concerns the target’s non-cooperativity and the second the estimation of the clutter covariance matrix. Relatively to the first issue, it should be mentioned that Equation (39) allows simultaneous clutter suppression and target imaging through the range Doppler algorithm. It is clear that both the platform motion and the target’s own motion must be compensated by the reference vector ${\tilde{G}}_{D}\left(n,m_{\nu }\right)$ to obtain a focused image of the moving target. However, a full knowledge of such a reference vector does not represent a realistic scenario since the target’s motions are not know. The platform motion can be known and then can be compensated. A well-focused image of a non-cooperative target can be achieved by ISAR processing applied at the output of the SDAP filtering operation. As stated previously, ISAR processing must be applied individually to each detected target in order to be effective.

**Figure 6:**
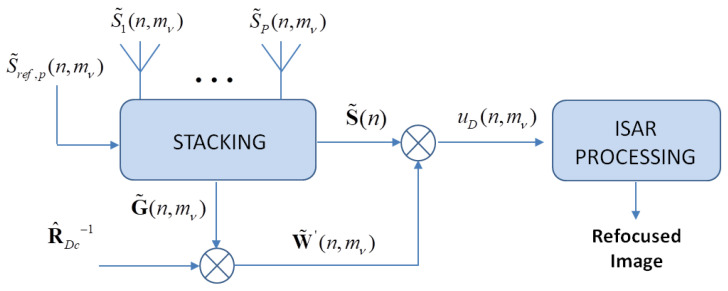
Optimum SDAP ISAR functional block.

The functional block of the SDAP-ISAR algorithm is shown in Figure 6.

The second issue to be addressed concerns the clutter covariance matrix estimation, i.e., ${\hat{R}}_{Dc}$. The Reed-Mallet-Brennan (RMB) rule [49], indicates that $N_{r}=2$ MP target-free and identically distributed range cells are needed to accurately estimate the clutter covariance matrix. In fact, ref. [49] demonstrates that, in such conditions, the average performance loss is roughly 3 dB with respect to a perfect knowledge of the clutter covariance matrix. As an example, if we consider values of PRF=2 KHz, $T_{obs}=0.5$ s and P=4, then $N_{r}=8000$ range cells would be needed to satisfy this condition. More practically, if we assume a range resolution of 0.5 m, this would mean that an area of 4 km in the range dimension where a homogeneous clutter should be present. It is quite easy to figure out that this condition cannot always be met in practical scenarios. In the next section, a sub-optimum approach will be presented to overcome this issue by reducing the dimension of the clutter covariance matrix, which, in the Doppler domain, is termed cross-power spectral matrix.

### 3.3. Use Case—SDAP-ISAR

The SDAP-ISAR algorithm presented in the previous section has been tested on real data to prove the effectiveness of SDAP-ISAR in terms of joint clutter suppression and target imaging. The measurement campaign took place on 18 July 2018 close to Teuge airport, in the Netherlands. The radar system used for the acquisitions is characterised by one transmitter and four receiving channels at X-band. Both the FMCW SAR system and the navigation unit (GNSS-IMU) were installed and operated on board of Cessna 208. The acquisition and the radar parameters are briefly summarised in Table 1.

The baseline between adjacent channels ($b_{l}=0.08$ m) is quite long and, by considering the imaging area size, namely $D_{y1}$, does not meet the condition imposed by Equation (12). In fact, by looking at the parameters shown in Table 1, the cross-range image size can be roughly evaluated by considering the antenna azimuth aperture and the slant range distance, i.e.,

(41) $$D_{y1}\approx R_{0}\theta_{az}=603m$$

**Figure 7:**
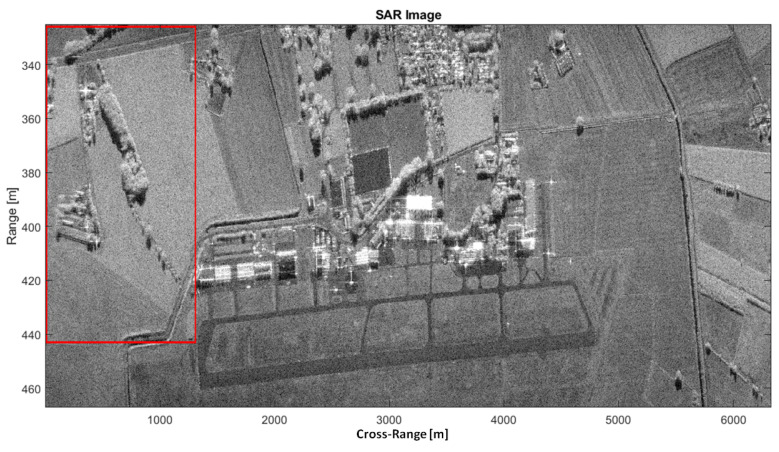
SAR image of the observed area formed via the range Doppler algorithm (RDA). The red box include the area of interest.

**Figure 8:**
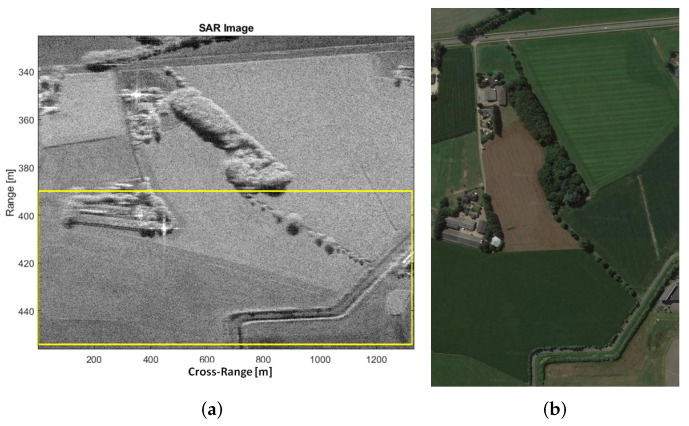
Image of the area under test (**a**) RDA SAR image—the yellow box includes the training area used for the clutter covariance matrix estimation, (**b**) Optical Google image of the area under test.

The array size is therefore too large for the multichannel range-Doppler to be applied. In fact, distortions appear that are induced by the $J(y_{1})$ term. To coherently sum the range-Doppler images, it is possible to virtually reduce the baseline between two adjacent channels. To this purpose, the first $N_{d}$ samples are discarded in channel 1 and the last $N_{d}$ samples are discarded in channel 2. In this way, the equivalent baseline $b_{l,eq}$ between two adjacent channels becomes $b_{l,eq}=b_{l}-N_{d}v_{p}T_{R}$, where $v_{p}$ and $T_{R}$ are the platform velocity and the Pulse Repetition Interval (PRI), respectively. It is worth pointing out that an additional temporal decorrelation is introduced because, after discarding those samples, the measurements are not longer simultaneous. It should be mentioned that this is not an issue for the image formation processing but it can affect the performance of clutter suppression. In order to appropriately select the training cells for clutter covariance matrix estimation, an accurate SAR image formation of the observed area is needed. The SAR image can be formed via a two-dimensional compression of the received signal. Typically, the main differences between SAR reconstruction algorithms consist of the way the Range Cell Migration Compensation (RCMC) and azimuth compression are handled. In this paper, the range-Doppler Algorithm (RDA) is taken into account. As often occur in practice, during real experiments, a misalignment between the true position of the SAR platform and the position measured by the IMU system may be experienced. As a consequence, a residual range migration may still be presents after a nominal RCMC. The SAR image of the area around the Teuge airport is shown in Figure 7, where the red box includes the area of interest. The red box is better shown Figure 8a while an optical Google image of the same area is shown in Figure 8b. The observation time and the corresponding cross-range resolution are detailed below:

(42) $$\begin{array}{c} T_{obs}=\frac{L}{v_{p}}=13.4\;s\\ \delta_{az}=\frac{cR_{0}}{2f_{0}v_{p}T_{obs}}=0.045\;m \end{array}$$

It is worth pointing out that a despeckle filter, namely the Lee filter, is applied after the RDA processing. After SAR image formation, the clutter covariance matrix must be estimated by using some training data. It is worth pointing out that the SDAP is computationally burdensome and the use of a standard PC may not be sufficient. As a matter of fact, a large synthetic aperture, i.e., $\theta_{az}\approx 20^{\circ }$, imposes to process a high number of samples, which can be calculated as follows:

(43) $$N_{samp}=T_{obs}PRF\approx 39000$$

**Figure 9:**
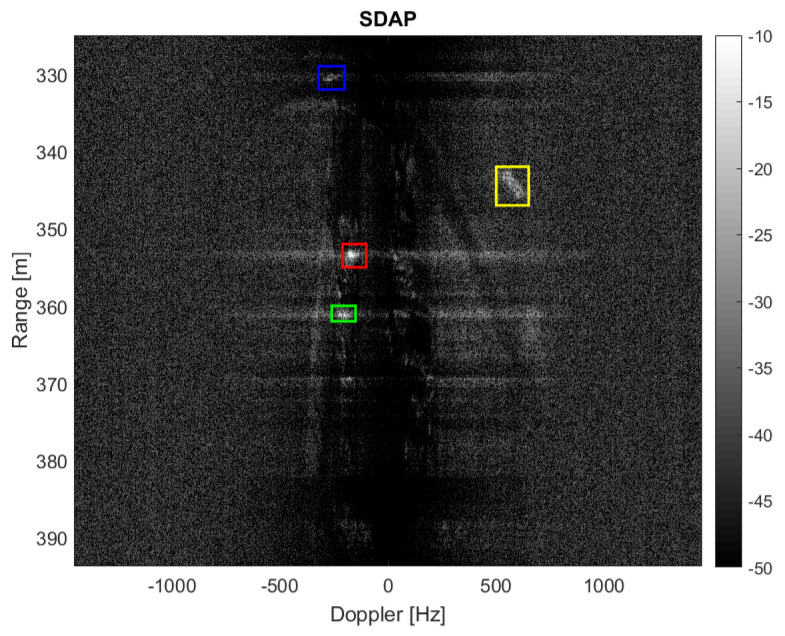
SAR image after clutter suppression via SDAP in which the detected targets are highlighted in the yellow, blue, green and red boxes. A smaller number of available slow-time samples is exploited since SDAP is computationally burdensome when a standard PC is used.

A reduced number of samples, i.e., $N_{samp}\approx 2000$, will be considered here to be able to handle the data with a simple workstation. It is clear that a reduced number of samples degrades the SAR azimuth resolution, therefore producing a worse range-Doppler image after applying the SDAP technique. However, the application of SDAP for clutter suppression is possible. Although a reduced number of samples is considered, a large amount of training data is required in order to estimate the clutter covariance matrix. In particular a number equal to $N_{r}=2N_{samp}P=16,000$ is needed, where P=4 is the number of available channels. This corresponds to an area of $\Delta_{r}=N_{r}\delta_{r}=4000$ m where $\delta_{r}=\frac{c}{2B}=0.25$ m is the range resolution. Therefore, the sub-optimum implementation of the SDAP algorithm is considered. In particular, a window length of L=30 is selected, which reduces the required training data to $N_{r}=2LP=240$. The area used to estimate the training data ranges from 390 m to 455 m and is highlighted by the yellow box in Figure 8a. The SAR image, after the application of the SDAP algorithm, is shown in Figure 9.

**Figure 10:**
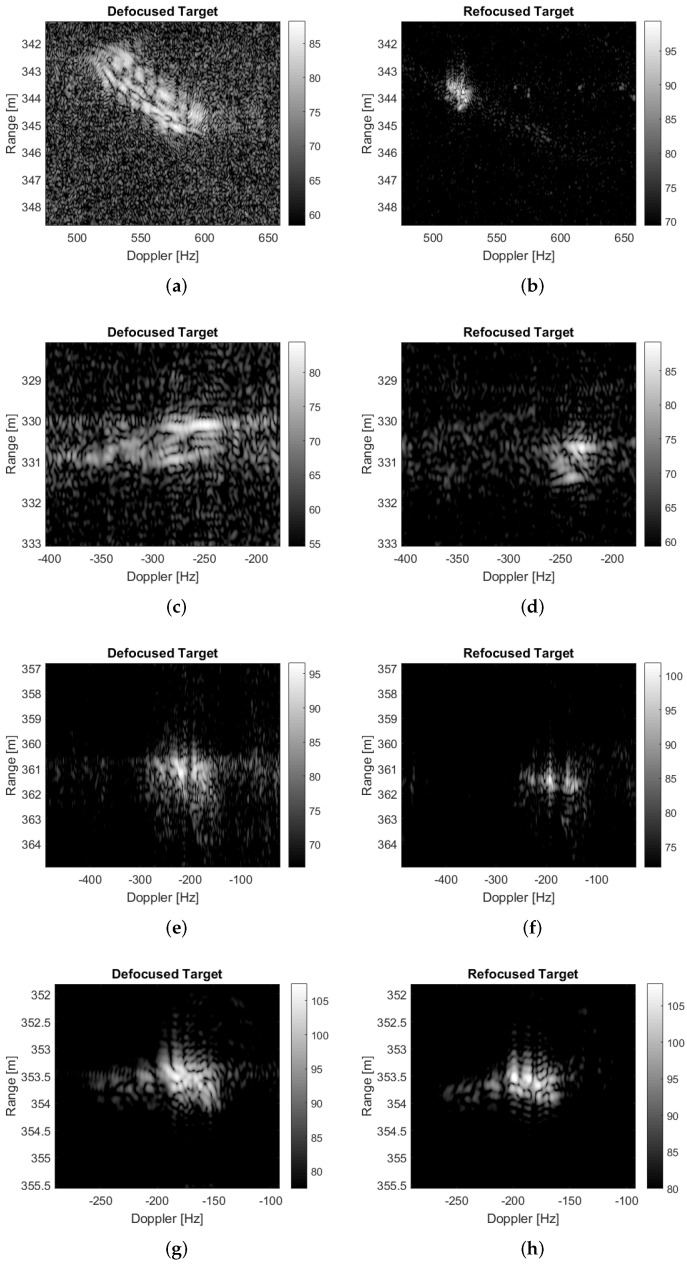
Target refocus through ISAR processing of Target 1 (yellow box in Figure 9) (**a**,**b**), Target 2 (blue box in Figure 9) (**c**,**d**), Target 3 (green box in Figure 9) (**e**,**f**) and Target 4 (red box in Figure 9) (**g**,**h**), respectively. (**a**,**c**,**e**,**g**) Before ISAR, (**b**,**d**,**f**,**h**) After ISAR.

Figure 9 clearly shows that the majority of the clutter has been suppressed and four targets, which have been highlighted in the yellow, blue, green and red boxes, can be easily detected. It should be mentioned that no specific technique has been used to select the training cells. Therefore, we cannot exclude that some outliers may be present within the selected training data, which, in turn, may degrade the clutter covariance matrix estimation and so the SDAP performance. Another aspect to be considered is that the ground truth is not available for this dataset and the clutter suppression performance cannot be assessed directly. This means that it is not possible to know whether the detected targets are actual moving targets or some residual stationary clutter. However, as previously described, ISAR processing can be exploited in this sense. Since each target has its own motion, to effectively apply ISAR processing, it must be separated from both the contributions of the static scene and from other targets. The detected targets depicted in yellow, blue, green and red boxes are shown, respectively, before and after the application of ISAR processing in Figure 10.

The improvement of the image focus is quite evident also from a visual point of view. This is true for the first three targets while there is no improvement for the fourth detected target. This means that the first three detected targets are most likely moving targets, for which the radial motion can be compensated, whereas the last one probably correspond to a fixed structure residual image (quite likely a house near a secondary road, which has a strong return and is not well suppressed by the SDAP algorithm). The improvement in the image focus can be also evaluated by looking at the Image Contrast (IC), which cab be calculated before and after the application of ISAR processing. The IC can be defined as follows:

(44) $$IC=\frac{\sqrt{E\left\{I-E\left[I\right]\right\}}}{E\left\{I\right\}}$$

where $E\left\{.\right\}$ denotes the average operation and *I* is the ISAR image magnitude. IC results before $\left(IC_{b}\right)$ and after $\left(IC_{a}\right)$ ISAR processing are shown in Table 2 for the four detected targets. Some additional considerations can be made regarding the radial velocity. In fact, the radial velocity can be expressed as $v_{r}=\frac{f_{D}\lambda }{2}$, where $f_{D}$ is the Doppler frequency. As the ISAR processor estimates the target’s radial velocity to compensate for the radial motion before forming the image, this can used as an additional information about the target.

**Figure 11:**
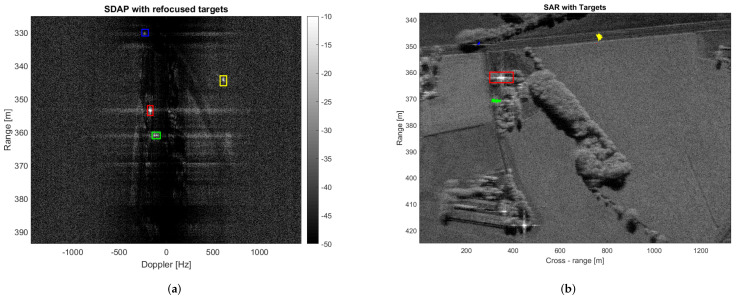
SAR images with refocused targets. (**a**) SAR image with a reduced number of samples after SDAP. (**b**) RDA SAR image with a superimposed refocused targets.

**Figure 12:**
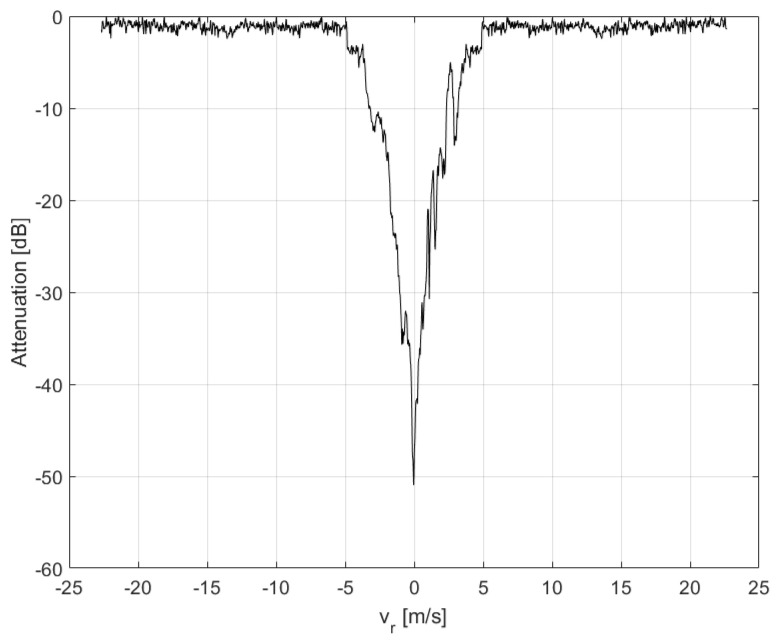
SDAP filter in the radial velocity domain.

The refocused moving targets are shown in Figure 11a after having been replaced in the range-Doppler SAR image obtained after applying the SDAP filter, whereas, the same refocused targets are shown in Figure 11b after having been superimposed to the original RDA SAR image. In the latter, the blue, green and yellow dots indicate the moving targets whereas the stationary structure is highlighted within the red box. The performance, in terms of clutter suppression, can be evaluated by displaying the clutter attenuation as a function of the radial velocity. This function can be seen as a filter in the radial velocity domain, as shown in Figure 12. In general, the filter notch is expected to be centred in the Doppler frequency of the focusing point. For a stripmap and non-squinted SAR, as it is for the case at hand, this corresponds to the zero Doppler frequency. Moreover, the Doppler null bandwidth is linked to the clutter covariance matrix estimation accuracy and to the number of spatial degrees of freedom, i.e., the number of channels. In this case, an adequate level of clutter suppression can be achieved and moving targets with a radial velocity greater than 2.5 m/s can be detected.

## 4. Virtual SDAP

In practical cases, it would be convenient and more economic to realise a single channel system rather than a more complex and costly multichannel one. Moreover, calibration issues typically arise when multi-channel systems are used, which degrade the overall performances of multi-channel signal processing, including SDAP. In this section, we introduce the concept and implementation of a virtual multi-channel system, which in turns enables SDAP processing.

### 4.1. Signal Model

**Figure 13:**
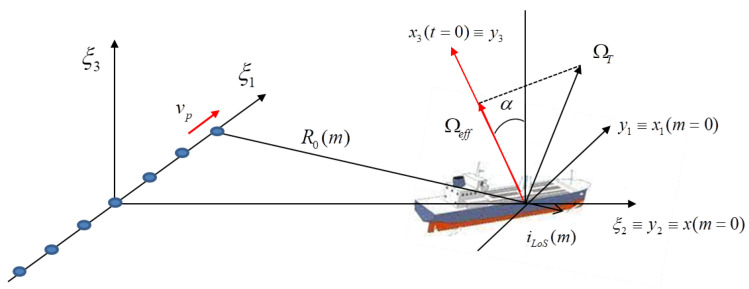
Acquisition geometry with a multichannel side-looking SAR system.

**Figure 14:**
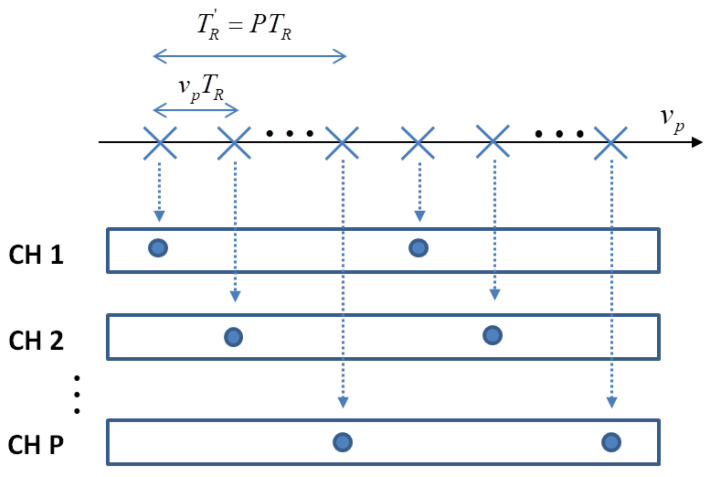
Data rearrangement.

Under the same geometrical configuration described in Section 2.1 and with reference to Figure 13, the discrete time-frequency model of the received signal can be represented as above:

(45) $$\begin{array}{cc} S\left(n,m\right) & ≐S\left(f_{0}+n\Delta f,mT_{R}\right)\\ & =S_{t}\left(n,m\right)+S_{c}\left(n,m\right)+N\left(n,m\right) \end{array}$$

where, as said previously, $S_{t}\left(n,m\right)$ is the target signal component, $S_{c}\left(n,m\right)$ represent the static scene return and N(n,m) is the additive noise. The indexes $m=\left[-\frac{M}{2},...,\frac{M}{2}-1\right]$ and $n=\left[-\frac{N}{2},...,\frac{N}{2}-1\right]$ represent, respectively, the pulse number and frequency, whereas $T_{R}=\frac{1}{PRF}$ is the Pulse Repetition Interval (PRI) and Δf is the frequency sampling step. It is worth reminding that the PRF determines the Doppler non-ambiguous region. Typically, the PRF is suitably chosen to be as large as the maximum static scene Doppler bandwidth. This avoids any image distortion due to Doppler folding. In some cases, the PRF may be selected to be significantly higher than the Doppler occupancy of the static clutter, such as in the case of ground moving target indication, where the target’s velocity may induce Doppler frequencies that are significantly higher than those produced by the static clutter. Under the assumption of a PRF significantly higher than the Doppler occupancy of the static clutter, i.e., $\left(PRF>B_{D}\right)$, the received signal may be subsampled without introducing any distortion in the SAR image. A multi-channel system can be emulated by rearranging the acquired data by introducing a sub-sampling operation in the slow time domain, as depicted in Figure 14. With reference to Figure 14, the first sample collected by the system can be thought of as being acquired by the first virtual channel, the second sample by the second virtual channel and so on. Considering the *p*th virtual channel, the received signal can be written as in Equation (46):

(46) $$\begin{array}{cc} S_{p}\left(n,m^{\prime }\right) & ≐S_{p}\left(f_{0}+n\Delta f,m^{\prime }T_{R}^{\prime }\right)\\ & =S\left(f_{0}+n\Delta f,m^{\prime }T_{R}^{\prime }+pT_{R}\right) \end{array}$$

where $m^{\prime }=\left[-\frac{M^{\prime }}{2},...,\frac{M^{\prime }}{2}-1\right]$ is the pulse index, p=[0,...,P−1] represent the index of the virtual channel while $M^{\prime }=\frac{M}{P}$ is the number of pulses and $T_{R}^{\prime }=P\cdot T_{R}$ is the PRI, which is the same for each virtual channel. It must be mentioned that the original value of the non-ambiguous Doppler region is lowered for each virtual channel. The non-ambiguous Doppler region for each virtual channel can be calculated by posing the reduced value equal to $PRF^{\prime }=\frac{PRF}{P}$. It should also be noted that the samples are not collected simultaneously across the virtual channels as it happens in an actual multichannel SAR system. The non-simultaneous acquisition effect must be taken into account in the signal modelling. More specifically, we will consider the effects of non-simultaneous acquisition among virtual channels in the statistical description of the clutter contribution.

### 4.2. Clutter Component

With regard to the clutter component, namely $S_{c,p}\left(n,m^{\prime }\right)$, we will refer to the model introduced in [15,50], which can be modified to account the non-synchronous acquisition across the virtual channels, as detailed in [33]. In particular, the clutter space-time covariance matrix, namely R, can be expressed as

(47) $$\begin{array}{cc} E & \left\{S_{c,p}\left(n,m\right)S_{c,q}^{*}\left(n,l\right)\right\}\\ & =P_{c}\rho_{s}\left[\left(l-m\right)v_{p}T_{R}^{\prime }+\left(p-q\right)v_{p}T_{R}\right]\\ & \times \rho_{t}\left[\left(l-m\right)T_{R}^{\prime }+\left(p-q\right)T_{R}\right] \end{array}$$

where $E\left\{\cdot \right\}$ expresses the expectation operator, $\left(l,m\right)$ the pulse indexes, $\left(p,q\right)$ the virtual channel indexes, $P_{c}$ is the clutter power, $\rho_{s}\left(\Delta \xi \right)=e^{-\frac{\Delta \xi^{2}}{2\sigma_{s}^{2}}}$ the spatial correlation coefficient and $\rho_{t}\left(\Delta t\right)=e^{-\frac{\Delta t^{2}}{2\sigma_{t}^{2}}}$ the temporal correlation coefficient.

### 4.3. Remarks

Equation (47) and Figure 14 lead us to make some considerations.

- The virtual M-SAR baseline, $d=v_{p}T_{R}$, and the virtual array size, $D=Pv_{p}T_{R}$, depend on the radar PRI and the platform velocity. Both those parameters can be set without taking into account the antenna physical size. Moreover, these same parameters allow for the term $J\left(y_{1}\right)$ to be controlled.
- The non-simultaneous acquisition across the *P* virtual channels, which is taken into account by the term $\left(p-q\right)T_{R}$ in Equation (47), can be often ignored. In fact, in the case of stationary ground clutter, the time decorrelation can be reasonably neglected, which makes the clutter statistical description substantially identical to that of a physical M-SAR systems.
- The price to be paid for the realisation of a virtual multi-channel radar system is the reduction of the non-ambiguous Doppler region with respect to the original single channel system. Therefore, in order to form virtual channels without introducing any Doppler ambiguity over the stationary clutter bandwidth, the system PRF should be suitably chosen.

With respect to the last remark, in order to avoid Doppler ambiguities, the following condition must be met:

(48) $$PRF^{\prime }=\frac{PRF}{P}\geq B_{D}=\frac{2D_{y_{1}}f_{0}v_{p}}{cR_{0}}$$

where $D_{y_{1}}$ is the size of the illuminated area along the cross-range dimension. It is worth reminding that a maximum value for the PRF must also be considered to avoid range ambiguities. This can be set as follows:

(49) $$PRF\leq \frac{c}{2D_{y_{2}}}$$

where $D_{y_{2}}$ is the size of the illuminated swath along the range dimension. In conclusion, both conditions in (48) and (49) must be satisfied.

### 4.4. Clutter Suppression and Imaging

If a virtual M-SAR can be enabled, Space Doppler Adaptive Processing (SDAP) can be applied. A slightly different notation will be introduced in this subsection without entering into the fine details of SDAP, as this has been detailed in Section 3. The received signal vector in the space Doppler domain is defined as

(50) $$\begin{array}{cc} \tilde{S}\left(n\right) & ={\left[\tilde{S}\left(n,0\right),\tilde{S}\left(n,1\right),...,\tilde{S}\left(n,M^{\prime }-1\right)\right]}^{T}\\ & \in C^{M^{\prime }P\times 1} \end{array}$$

where

(51) $$\begin{array}{cc} \tilde{S}\left(n,m_{\nu }\right) & =\frac{1}{P}{\left[{\tilde{S}}_{1}\left(n,m_{\nu }\right),{\tilde{S}}_{2}\left(n,m_{\nu }\right),...,{\tilde{S}}_{P}\left(n,m_{\nu }\right)\right]}^{T}\\ & \in C^{P\times 1} \end{array}$$

(52) $${\tilde{S}}_{p}\left(n,m_{\nu }\right)=DFT_{m^{\prime }}\left\{S_{p}\left(n,m^{\prime }\right)\right\}$$

and $m_{\nu }$ is the Doppler frequency index. The reference vector in the space-Doppler domain is expressed as

(53) $$\begin{array}{cc} \; & {\tilde{G}}_{D}\left(n,m_{\nu }\right)=\\ & {\left[{\tilde{S}}_{ref}\left(n,m_{\nu }\right),{\tilde{S}}_{ref}\left(n,m_{\nu }-1\right),...,{\tilde{S}}_{ref}\left(n,m_{\nu }-\left(M^{\prime }-1\right)\right)\right]}^{T}\\ & \in C^{M^{\prime }P\times 1} \end{array}$$

where

(54) $$\begin{array}{cc} \; & {\tilde{S}}_{ref}\left(n,m_{\nu }\right)=\\ & \frac{1}{P}{\left[{\tilde{S}}_{ref,1}\left(n,m_{\nu }\right),{\tilde{S}}_{ref,2}\left(n,m_{\nu }\right),...,{\tilde{S}}_{ref,P}\left(n,m_{\nu }\right)\right]}^{T}\\ & \in C^{P\times 1} \end{array}$$

and

(55) $${\tilde{S}}_{ref,p}\left(n,m_{\nu }\right)=DFT_{m^{\prime }}\left\{S_{ref,p}\left(n,m^{\prime }\right)\right\}$$

Since $M^{\prime }$ can be large, a sub-optimum approach can be implemented. With the use of the V-SDAP notation, this can be done by splitting the $M^{\prime }$ Doppler bins into sub-blocks of length *L* before carrying out the optimum cancelling filtering in each block and coherently summing the outputs, to produce:

(56) $$u_{D,w}\left(n,m_{\nu }\right)=\sum_{i}u_{D,i}\left(n,m_{\nu }\right)$$

where

(57) $$u_{D,i}\left(n,m_{\nu }\right)={\tilde{W}}_{D,i}^{H}\left(n,m_{\nu }\right){\tilde{S}}_{i}\left(n\right)$$

in which ${\tilde{W}}_{D,i}\left(n,m_{\nu }\right)$ is the weightvector with respect to the *i*th block expressed as

(58) $${\tilde{W}}_{D,i}\left(n,m_{\nu }\right)={\hat{R}}_{Dc,i}^{-1}{\tilde{G}}_{D,i}\left(n,m_{\nu }\right)$$

where ${\tilde{G}}_{D,i}\left(n,m_{\nu }\right)$, ${\tilde{S}}_{i}\left(n\right)$ and ${\hat{R}}_{Dc,i}$ are the reference vector, the signal vector and the cross-power spectral matrix in the *i*th block expressed as

(59) $${\tilde{G}}_{D,i}\left(n,m_{\nu }\right)=\left[\begin{array}{c} {\tilde{S}}_{ref}\left(n,m_{\nu }-\left(i-1\right)L\right)\\ {\tilde{S}}_{ref}\left(n,m_{\nu }-\left((i-1)L+1\right)\right)\\ {\tilde{S}}_{ref}\left(n,m_{\nu }-\left((i-1)L+2\right)\right)\\ \vdots \\ {\tilde{S}}_{ref}\left(n,m_{\nu }-\left(iL-1\right)\right) \end{array}\right]\in C^{LP\times 1}$$

(60) $${\tilde{S}}_{i}\left(n\right)=\left[\begin{array}{c} \tilde{S}\left(n,(i-1)L\right)\\ \tilde{S}\left(n,(i-1)L+1\right)\\ \tilde{S}\left(n,(i-1)L+2\right)\\ \vdots \\ \tilde{S}\left(n,iL-1\right) \end{array}\right]\in C^{LP\times 1}$$

and

(61) $${\hat{R}}_{Dc,i}=\frac{1}{N_{r}}\sum_{n_{r}=0}^{N_{r}-1}{\tilde{Z}}_{i}\left(n_{r}\right){\tilde{Z}}_{i}^{H}\left(n_{r}\right)\in C^{LP\times LP}$$

where ${\tilde{Z}}_{i}\left(n_{r}\right)$ represents the target-free echo, relatively to the $n_{r}$ range cell and to the *i*th window.

Equivalently to the case of physical SDAP-ISAR, a V-SDAP-ISAR processing can be enabled by applying an ISAR processor to the clutter removed SAR image for each of the detected moving targets.

### 4.5. Use Case—V-SDAP-ISAR

The Virtual V-SDAP (SDAP) algorithm is tested in this subsection by using real data acquired by using a two-channel SAR system with high PRF. The measurement campaign took place on the 5 June 2016 by flying over a highway and perpendicularly with respect to it. The acquisition and the radar parameters are briefly summarised in Table 3. Particularly, in this use case, the results obtained by applying V-SDAP are discussed and compared to those obtained by implementing a physical SDAP. More specifically, a virtual three-channel system is obtained by virtualising a single antenna and is then compared to a physical two-channel SDAP.

It should be noted that the system has a high PRF, when compared to the stationary clutter Doppler bandwidth, which allows for V-SDAP to be effectively applicable. However, the small transmitted bandwidth, i.e., B=120 MHz produces a poor slant-range resolution, i.e., $\delta_{r}=1.25$ m, which does not allow for a high range resolution over small targets, such as cars travelling on the highway. Nevertheless, with the aim of determining the effectiveness of the SDAP approach, we will concentrate on the ability of obtaining ISAR images of moving targets when immersed in strong ground clutter, regardless of the level of details that can be obtained by post-processing the ISAR images for target recognition purposes. Virtual SDAP exploits high PRFs to emulate a multichannel system. Under the hypothesis that the PRF is higher than the Doppler occupancy of the SAR scene, a sub sampling in the slow-time domain can be applied and samples can be rearranged to emulate virtual multichannel SAR. However, sub sampling involves a reduction of the non-ambiguous Doppler region with respect to the single channel SAR data. When the PRF is not higher enough than the clutter Doppler bandwidth, Doppler aliasing can occur. As a consequence, the clutter folds back and the SDAP filter performance degrades. In particular, the SDAP filter introduces a large bandwidth notch at those radial velocities where the clutter folds back. In order to avoid Doppler ambiguities, the condition in Equation (49) must be met. The cross-range imaging size can be roughly evaluated by considering the receiving antenna beamwidth, i.e., $D_{y1}\approx \theta_{az}R_{0}=261$ m. Therefore, the clutter Doppler bandwidth can be expressed as:

(62) $$B_{d}=\frac{2D_{y}f_{0}v_{p}}{cR_{0}}=417.6\;\mathrm{Hz}$$

**Figure 15:**
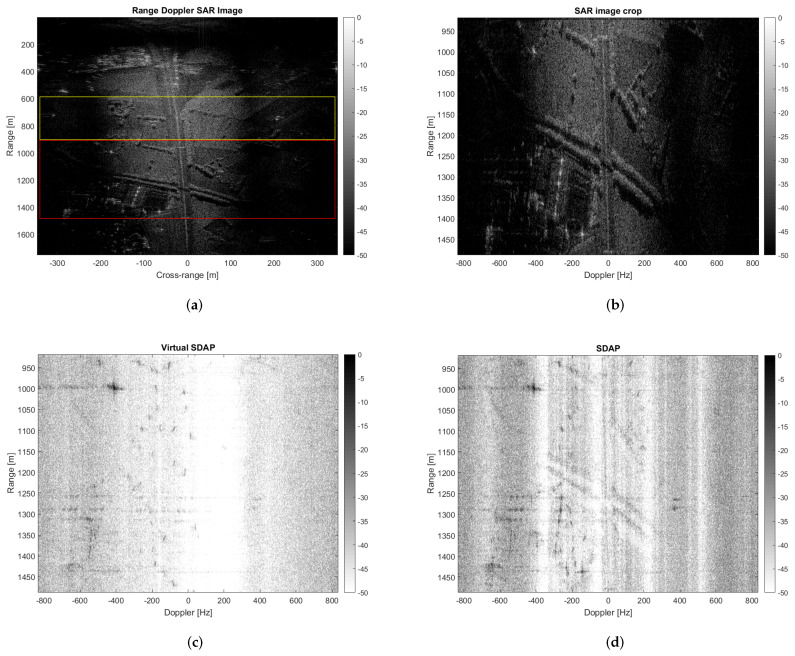
Clutter suppression results. (**a**) SAR image of the observed area. The area under test is included in the red box while the training area is highlighted within the yellow box. (**b**) SAR image of the area under test. (**c**) SAR image after clutter suppression via virtual SDAP. (**d**) SAR image after clutter suppression via SDAP.

If three channels $\left(P^{{}^{\prime }}=3\right)$ are virtualised, the PRF lower bound is satisfied and V-SDAP can be applied without distortions. The dataset used in the previous section exploits a four-channel SAR system with a very low PRF, i.e., PRF=2.9 kHz and a larger antenna beamwidth than the current one, i.e., $\theta_{az}=20^{\circ }$. In this case the PRF constraint is not satisfied even if only two channels are virtualised. Figure 15a shows the multichannel range Doppler SAR image obtained by processing data acquired by one channel in such a way to synthesize $P^{{}^{\prime }}=3$ virtual channels. The clutter covariance matrix is estimated by using training data included in the yellow box while the red box highlights the region under test. Figure 15b,c show the image of the area under test before and after clutter suppression obtained by applying V-SDAP. The clutter-suppressed image obtained by applying a physical SDAP with two actual channels is shown in Figure 15d.

**Figure 16:**
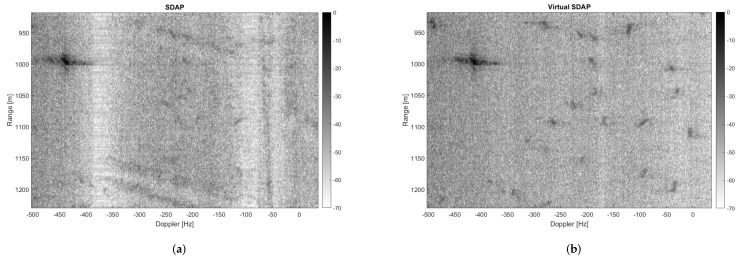
Zoom-in of the SAR image after clutter suppression. (**a**) Clutter suppression via conventional SDAP where two actual channels are employed. (**b**) Clutter suppression via virtual SDAP where three channels are virtualised and used.

**Figure 17:**
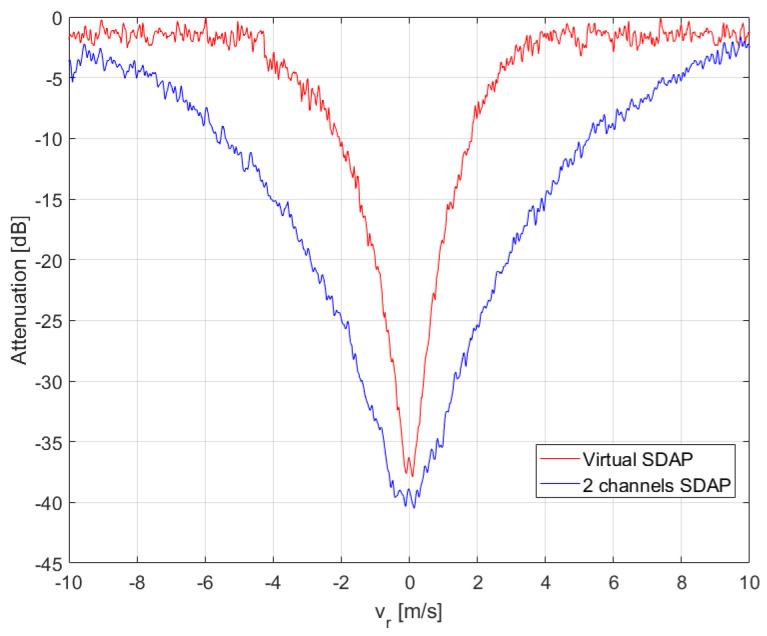
SDAP filter in the radial velocity domain. The blue trend represents the two-channel physical SDAP filter while the red trend represents the three-channel virtual SDAP filter. V-SDAP allows for a narrower filter bandwidth to be obtained and thus for targets with lower radial velocities to be detected.

Noticeably, the clutter is suppressed more effectively when using V-SDAP. This result can be justified with two main reasons. Firstly, the V-SDAP applied here creates thrual channels as opposed to the two physical channels that have been used for the physical SDAP, therefore incrementing the available spatial DoFs. Secondly, the use of a single physical channel avoids any cross-channel mis-calibration issues, which typically occur when dealing with multi-channel systems. To better asses clutter suppression and target detection performance, a crop of the observed area is shown in Figure 16. In particular, Figure 16a,b, show the results relative to SDAP, with two physical channels, and to V-SDAP, with thrual channels. A comparison in terms of radial velocity filtering is displayed in Figure 17. Noticeably, a larger number of channels (either physical or virtual) allows for the filter bandwidth to be reduced and, therefore, for targets with lower radial velocities to be detected. The example presented in this section shows how GMTImaging can be implemented effectively with a single channel system. Moreover, it can be observed that, when high PRFs can be used, a V-SDAP implementation may produce better performances than a more complex and costly two-channel system.

## 5. Cognitive Ground Moving Target Imaging

Modern radar systems are often demanded to have multiple functions, such as detection, imaging and classification, and to operate in heterogeneous and rapidly changing scenarios. Systems that operate in such conditions require a new architecture paradigm, which enables some level of system cognition. In this way, cognitive radar systems should be able to sense the environment and autonomously adapt to optimise their performance, also given the specific task that has been commanded. A cognitive radar system learns from past experience, which has been acquired by means of past actions and with a continuous interaction with the environment. More specifically, a cognitive radar optimally adapts its transmitted waveform (action) and its signal processing on receive (perception) based on the feedback received from the environment and also based on past experience (memory). The concept of cognitive radar was introduced for the first time by Simon Haykin in [51]. In parallel, in recent years, the Defense Advanced Research Projects Agency (DARPA) has been working on the development of a knowledge-aided adaptive radar architecture that integrates some knowledge of the environment into the adaptive space-time beamformer [52]. In addition to theoretical findings, the recent technology, including Software Defined Radio (SDR) technology, has matured enough to enable some preliminary development of cognitive radar systems. Parameters such as transmitted power, instantaneous bandwidth and PRF can be controlled automatically by a cognitive system in order to maximise the radar performances and the overall mission success. In this section, we will apply some basic concepts of cognitive radar to the problem of ground moving target imaging. More specifically, a cognitive C-SDAP (SDAP) will be defined and implemented to improve GMT Imaging performances. As amply discussed in this paper, for SDAP to be applied effectively, the space-time characteristics of the clutter statistics must be known or accurately estimated. In practical cases, an accurate estimation of the clutter covariance matrix is not a trivial step due to the likely presence of heterogeneous clutter and the lack of available training data. In more details, in this section, a high-level cognitive radar architecture will be defined and developed to optimise SDAP for GMT Imaging. Real data acquired with a multichannel airborne system will be used to assess performances and compare to the classic SDAP approach.

### 5.1. Rule-Based Cognitive Architecture

**Figure 18:**
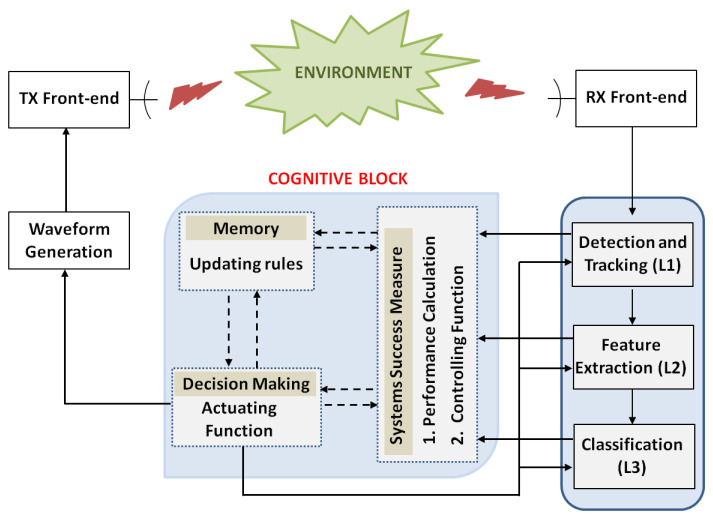
Rule-based cognitive radar architecture.

One way of implementing a cognitive system is through a set of adaptive rules. A rule-based cognitive architecture is typically defined through a set of performance metrics. Such metrics can be utilised to set the rules with which the radar system reacts to the environment feedback, even more importantly when a change is registered in the environment. Although this type of architecture will be defined and tested for GMT Imaging applications, it is generally applicable to more complex missions. The main blocks of the proposed cognitive rule-based architecture are shown in Figure 18 whereas a brief description is provided here below:

- Transmitter and receiver blocks. The transmitter adapts the transmitted waveform parameters to environmental changes in order to maintain a desired system performance. Performances are measured through a set of performance indexes, which are calculated on the received and processed signal. Cognition is applied be adopting a learning process, which is enabled through the interaction between the system and the environment and by using memory and measures of success.
- Signal processing block. It processes the received echoes according to the radar mission and the past experience. It is connected to the cognitive block with which it exchanges information and receives updated optimal parameters to achieve the desired performance for the specific radar mission.
- Cognitive block. The information extracted by the signal processing block is exploited to update the transmitting parameters. This process is based on a comparison between past and current performances, which ensures that the system learns from its past actions. The cognitive block includes three sub-blocks, namely the *System Success Measure*, *Memory* and *Decision Making* blocks. The fist one defines the rules, i.e., the controlling functions that account for external changes. Such rules are based on performance indexes, which are able to assess how the system reacts to the environment and to the stimuli produced by the transmitter. Each controlling function produces an output that is directly use to drive, through the actuating functions, the system’s response, which, in turn, updates the transmitting parameters. The memory keeps track of the changes that have been observed and, consequently, made by the system. The memory is a fundamental block that allows for the system to learn from its past actions. Finally, the decision making block updates transmitter’s parameters through to the actuating functions in order to optimise the system performances.

### 5.2. Cognitive Design for Moving Target Imaging

In this section, the rule-based cognitive approach that has been described in the previous sub-sections is applied to solve the problem of clutter suppression for the detection of moving targets in a heterogeneous and dynamically changing environment. The main steps of the signal processing implementation and the definition of the controlling and actuating functions are shown as follows.

#### 5.2.1. SAR Image Formation

The first step concerns the formation of a SAR image from the received data. This processing can be considered as a two-dimensional compression of the backscattered signals that aims at producing a high-resolution two-dimensional radar image. In this work, the range-Doppler Algorithm (RDA) has been considered for its simplicity. Other more accurate but more complex and computationally expensive algorithms may be used in its place [53].

#### 5.2.2. SAR Image Segmentation

SDAP processing is based on the estimation of the space-Doppler clutter statistics through the clutter covariance matrix. In the case of a heterogeneous environment, the clutter must be segmented into different classes, such as land, grass, urban areas, etc, for an accurate estimation of the clutter covariance matrix. As a matter of fact, any clutter suppression algorithm performance is related to the training data used to determinate the clutter statistics, which should be as similar as possible to those of the clutter that is present in the cell under test (where the target may be present). The clutter can be classified based on a number of statistical characteristics. One important characteristic is the texture, which is often utilised as a clutter classification feature. Among many techniques for clutter texture analysis, it is worth mentioning the 2D Wavelet transform (2D-WT), [54]. The energy distribution of the 2D-WT can be exploited as a feature to describe the image texture. A predefined set of clutter classes can be stored in the system memory in terms of their relative textures, which represent a priori information to be used to implement an image segmentation. Each image pixel is then classified based on its similarity with the a priori classes. If a pixel is too “distant” from any of the known classes, it is declared as “unclassified”. A segmentation process terminates successfully if a large majority of the pixels are classified. If the ratio between the numbers of unclassified pixels $P_{NC}$ and the number of classified pixels $P_{C}$ is smaller than a given threshold, the segmentation process can be considered satisfactory, otherwise it is declared unsatisfactory and additional clutter classes may be present in the scene that are not included in the a priori class list. The following procedure is implemented that allows for new classes to be added to the list of priors. Firstly the SAR image is divided in sub-blocks and the ratio between unclassified and classified pixels is calculated for each sub-block *b* as in Equation (63)

(63) $$Cl_{b}=\frac{P_{NC_{b}}}{P_{C_{b}}}$$

A corresponding controlling function is defined through the introduction of a threshold to identify the presence of a new class of clutter. Specifically,

(64) $$\alpha_{1,b}=\left\{\begin{array}{c} 0\;if\;Cl_{b}\leq \gamma_{c}\\ 1\;if\;Cl_{b}>\gamma_{c} \end{array}\right.$$

When the index $Cl_{b}$ exceeds a predefined threshold, the relative sub-block is considered not well-segmented and the presence of a new clutter class is declared. The corresponding feature vector (texture descriptor) can be obtained by applying the 2D-WT to the unclassified pixels and subsequently stored in the memory as a new clutter class. Then, $\alpha_{1,b}$ is used to update the memory. If at least one sub-block is found to be not well-segmented, the system performs a new segmentation by following the same procedure. The iteration stops when no more classes are identified.

(65) $$\gamma_{1}=\sum_{b}\alpha_{1,b}=\left\{\begin{array}{c} 0\\ >0\;retrain \end{array}\right.$$

#### 5.2.3. Training Data Selection

After having performed the image segmentation, a homogeneous clutter area of the same time as the clutter around the cell under test can be selected to be used as a training dataset. However, some outliers, such as other moving targets, may be present in the selected training set, which would degrade the SDAP performance. In order to detect the presence of outliers, a non-homogeneity detector (NHD) can be implemented and applied directly to the data. A method based on the generalised inner product (GIP) has been proposed that effectively detect the presence of outliers and consequently remove them from the training set [55]. Moreover, to account for the RMB rule, the number of selected training cells should be considered and compared to the threshold, namely 2MP. The corresponding controlling function can be defined as follows:

(66) $$\alpha_{2}=\left\{\begin{array}{c} 0\;if\;N_{r}<2MP\\ 1\;if\;N_{r}\geq 2MP \end{array}\right.$$

When $\alpha_{2}=0$, the system resort to using a predefined clutter covariance matrix, $M_{c}$, stored in the memory, which has the same statistical properties of the observed clutter. This can be jointly combined, via a Bayesian approach, with the estimated covariance matrix, [56]. The controlling function that defines this rule is shown in Equation (67)

(67) $$\alpha_{3}=\left\{\begin{array}{c} 0\;if\;M_{c}=0\\ 1\;if\;M_{c}=1 \end{array}\right.$$

where the variable $\alpha_{3}$ indicates the presence or absence of a clutter covariance matrix in the system memory that has similar characteristics to the clutter under test. Specifically, $\alpha_{3}=0$ indicates that no covariance matrix is stored in the memory and that the above mentioned technique cannot be used. In this latter case, a new acquisition is requested with a larger transmitted waveform bandwidth through the actuating function $\gamma_{2}=+1$. As a larger bandwidth improves the range resolution, a larger amount of clutter cells becomes available that can be used to satisfy the RMB rule. The relative actuating function can be expressed as follows:

(68) $$\gamma_{2}=\left\{\begin{array}{c} +1\;if\;\left(\alpha_{2}+\alpha_{3}\right)=0\;\\ 0\;otherwise \end{array}\right.$$

#### 5.2.4. Clutter Suppression and Target Detection

After selecting the training data and, consequently, estimating the clutter covariance matrix, the obtained SDAP filter can be applied to the received signal for clutter suppression. The controlling function $\alpha_{4}$ in Equation (69) measures the SDAP filter performance and compares it to the ideal Doppler (radial velocity) filter response. In particular, it accounts for both the position and the bandwidth of the SDAP filter notch in the radial velocity domain. In fact, it is expected that the filter notch, i.e., the Doppler null DN, is located in the Doppler frequency of the SAR scene centre (typically referred to as Doppler centroid). For a stripmap and non-squinted SAR, this value corresponds to the zero Doppler frequency. The Doppler null bandwidth DNB is instead linked to the estimation of the clutter covariance matrix and to the number of spatial degrees of freedom, i.e., the number of channels. If the clutter is suppressed correctly, the resulting Doppler filter should have a narrow bandwidth and should be centred around the Doppler centroid. The $DNB_{r}$ can be defined as the ratio between the notch bandwidth of the obtained Doppler profile and the notch bandwidth of the ideal one, whereas the $DN_{d}$ represents the difference between the Doppler null of the actual filter and that of the ideal one. The value of $DN_{d}$ is then compared to a threshold that sets the maximum allowed difference. When the difference between the actual and ideal filter in terms of clutter suppression exceeds the maximum value, the corresponding controlling function is set to $\alpha_{4}=1$, otherwise it is set to $\alpha_{4}=0$.

(69) $$\alpha_{4}=\left\{\begin{array}{c} 0\;if\;DN_{d}\leq \lambda_{DN}\;\&\;DNB_{r}\leq \lambda_{DNB}\\ 1\;\;otherwise \end{array}\right.$$

A value of $\alpha_{4}=1$ indicates that the STAP filter does not perform effectively. This may be due to the presence of some interferences in the signal bandwidth or to an insufficient number of spatial channels. The first case scenario may be solved by means of a spectrum sensing technique, which aims at varying the transmitted waveform in order to avoid the utilisation of the interfered part of the spectrum. This may be simply done my reducing the transmitted bandwidth to avoid the interference. If This is $F_{SS}$ is defined as a controlling function that assess the presence of interference in the signal bandwidth, the following actuating function can be implemented

(70) $$\gamma_{3}=\left\{\begin{array}{c} -1\;if\;F_{SS}=1\;\\ +1\;if\;F_{SS}=0 \end{array}\right.$$

The second case scenario would lead to the request of additional spatial channels, if available. In the following section, a use case is presented that outlines a specific scenario and analyses the positive effects of implementing a C-SDAP when compared against a standard SDAP.

### 5.3. Use Case—Cognitive SDAP-ISAR

**Figure 19:**
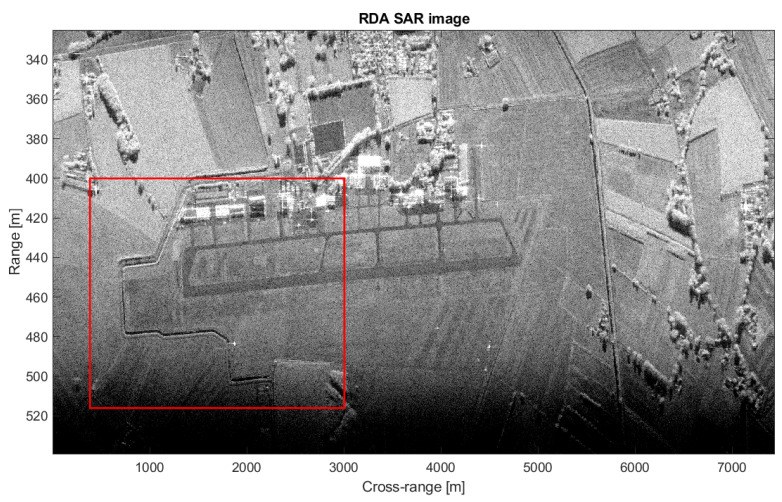
RDA SAR image of the observed area. The red box highlights the area under test.

This use case will show some results relatively to the use of C-SDAP. The dataset that has been used for this use case is the same that was used in Section 3.3 with the acquisition parameters as shown in Table 1. The observed area is displayed in Figure 7. In this use case, the cognitive approach has been tested on a different area of the same SAR image, which is contained in the red box, as shown in Figure 19.

**Figure 20:**
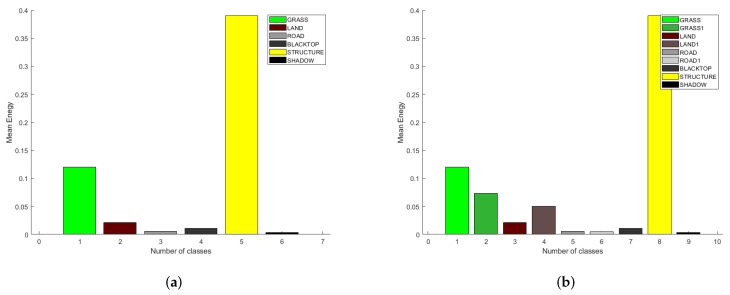
A representation of the content of the system memory. (**a**) initial memory content in which only a priori information are present. (**b**) the memory content after the image has been divided into sub-blocks: New clutter classes have been detected.

In all the previous implementations of SDAP, both physical and virtual, the training cell for the clutter covariance matrix estimation were selected randomly, i.e., without any specific criterion. Nevertheless, in order to perform an effective clutter cancellation, the training cells should be chosen appropriately and not randomly. This should be done to make sure that homogeneous clutter regions are selected and in order to avoid selecting cells where other targets are present. To this end, a segmentation of the SAR image is firstly performed. The segmentation of the SAR image is achieved by means of a 2D Wavelet transform (WT). The 2D-WT decomposes the image in four sub-images and the energy of each sub-image can be exploited as a specific feature for image segmentation. Each image pixel is classified based on the minimum Euclidean distance between the considered feature vector and a set of template vectors, which represent different classes of clutter. The set of template vectors are stored in the system memory and represents the a priori information. A priori information can be obtained by segmenting another area of the same image where main classes of clutter, i.e., grass, road, structures, ecc are presents. In this case, the selected area is close to the aerodrome de Spa La Sauveniere (NL). A representation of the a priori information that is stored in the system memory is shown in Figure 20a, where the mean value of the energy for each class of clutter is depicted. It is worth pointing out that the mean values of the energy remains approximately the same regardless of the section of the SAR image considered.

**Figure 21:**
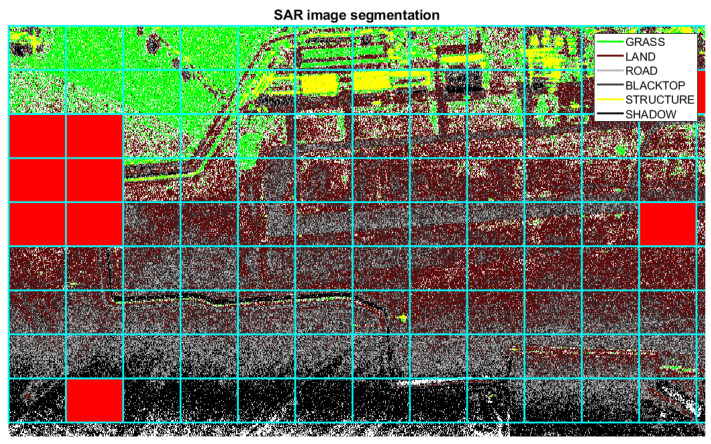
Performance evaluation of the SAR image after image segmentation: SAR is divided into sub-blocks to detect the presence of an additional classes not stored in the system memory. The sub-blocks containing new classes are highlighted in red.

The segmentation result of the SAR area under test is shown in Figure 21. The white pixels represent pixels that are not assigned to any class of clutter that is currently stored in the memory. In the segmented SAR image, it is however possible to recognise the airport runway and some structures adjacent to the runway. Based on the evaluation of the segmented image, the system tries to detect additional classes that are present in the image in order to improve the accuracy and variety of the memory content. After image segmentation, the system splits the image into sub-blocks and calculates the number of unclassified pixel and the number of classified pixel for each of them, in order to evaluate the performance index expressed in Equation (63) The considered sub-blocks are shown with cyan lines in Figure 21. If the $Cl_{b}$ index exceeds a predefined threshold, an additional type of clutter, not previously stored in the system memory, may be present in the considered sub-block. As a consequence, the corresponding feature vector can be extracted through the 2D-WT and stored in the system memory. The sub-blocks that exceeds the predetermined threshold are highlighted in red in Figure 21.

**Figure 22:**
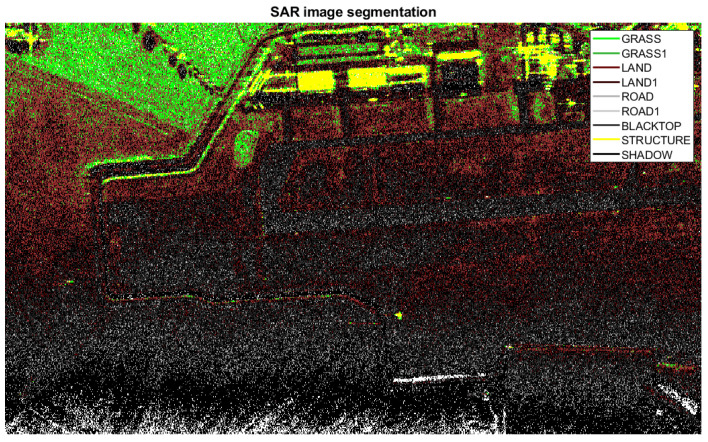
Segmentaed SAR image after a new segmentation step in which the memory has been updated with new textures belonging to new classes of clutter.

The updated memory is shown in Figure 20b, where new classes of clutter are present. A new segmentation is performed with the new a priori information. The result after the new segmentation is depicted in Figure 22.

**Figure 23:**
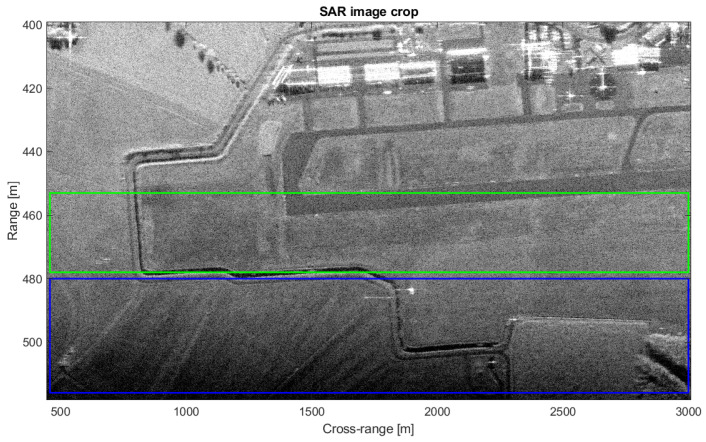
SAR image under test in which the training area is included in the green box, while the area on which to apply SDAP is included in the blue box.

If, after performing the new segmentation, the controlling function $\alpha_{1,b}$ is equal to zero for each sub-blocks, i.e., $\gamma_{1}=0$, then, the memory content is not further updated and no additional segmentations are performed. After the image segmentation, the training area can be selected more carefully in order to consider a homogeneous clutter. The structures and the airport runway, which have clutter statistics that different from the area under test can be avoided. Range cells that go from 455 m to 480 m are selected for the clutter covariance matrix estimation, as shown in Figure 23, where the training are is included in the green box and the test area in the blue box.

**Figure 24:**
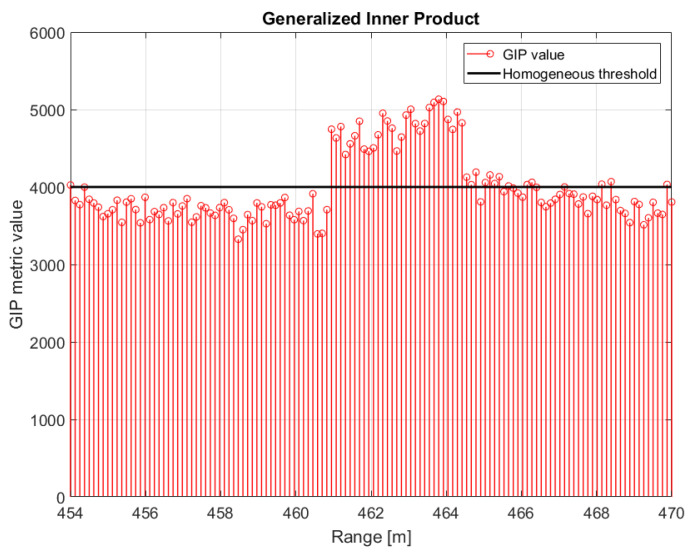
Result of the GIP test applied on the training data set.

However, some outliers may be present in the training area, which may degrade the clutter covariance matrix estimation. A Generalised Inner Product (GIP) test can be employed at this stage to excise such non-homogeneities in the training data set [55]. In order to validate the GIP test, a dummy target with a radial velocity of 3 m/s has been included in the data around 462 m. The GIP test result in shown in Figure 24 where the black line represent the homogeneity threshold. Heterogeneous data, such as outliers or other targets, can be detected and excluded from training set before estimating the covariance matrix and therefore improve the estimation accuracy.

**Figure 25:**
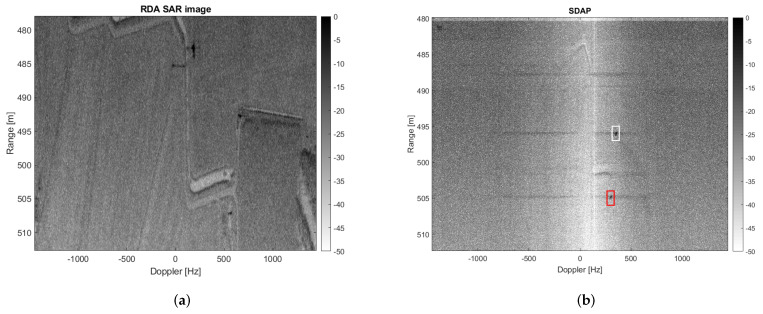
Result of the cognitive SDAP processing (**a**) Original SAR image of the area under test before clutter suppression, (**b**) SAR image after clutter suppression through SDAP.

Furthermore, in this case, too much training data is required to perform SDAP processing and sub-optimum approach is required. in this case a windows length of L=10 is considered and the required training data to perform sub-optimum SDAP is $N_{required}=80$. The initially selected training interval is from 455 m to 480 m correspond to $N_{r}=100$ since the range resolution is $\delta_{r}=\frac{c}{2B}=0.25$ m. However, the presence of non-homogeneous data reduce the training set to $N_{r}=60$. Since $N_{r}<N_{required}$, the controlling function $\alpha_{2}=0$. Therefore, the system try to improve the performance by applying the Bayesian SDAP approach by combining the estimated clutter covariance matrix with a clutter covariance matrix store in the system memory. More details about Bayesian approach can be found in [56]. In this case the cognitive chain does not require the system to increase the bandwidth and then $\gamma_{2}=0$. Results before and after clutter suppression are shown in Figure 25.

**Figure 26:**
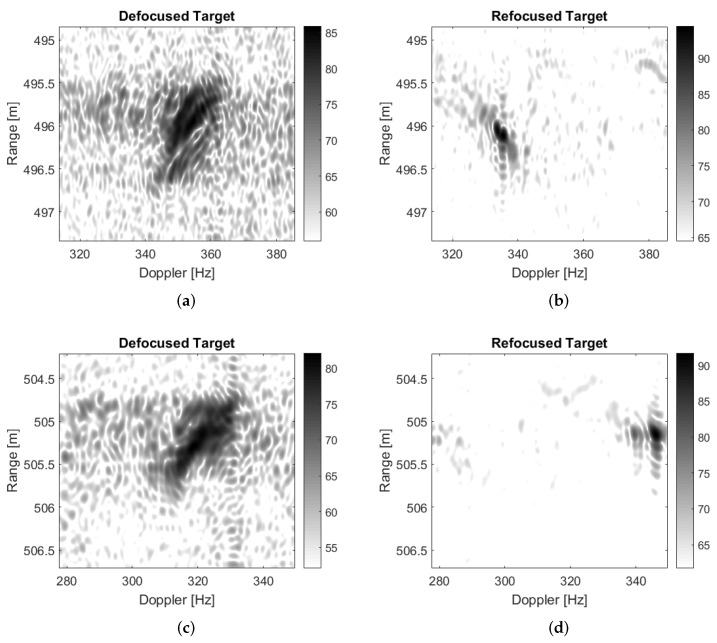
Refocusing through ISAR processing of Target 1 (**a**) before ISAR, (**b**) after ISAR and of Target 2 (**c**) before ISAR, (**d**) after ISAR.

It is worth pointing out that, as explained in Section 3.3, a reduced number of slow-time samples are considered, i.e., $N_{samp}\approx 2000$, since full SDAP is too burdensome for a standard PC. However, the clutter seems to be well suppressed and two moving targets, which are enclosed in the red and white boxes, can be easily detected. Furthermore, in this case, the ground truth is not available and, therefore, it is not possible to know if the detected objects are real moving targets or residuals of fixed structures that have not been suppressed sufficiently. However, moving targets appear defocused in SAR images. ISAR processing can be used to compensate the relative motion between moving targets and the SAR platform and, therefore, to refocus moving targets in SAR image. Detected targets, before and after ISAR processing, are shown in Figure 26a–d. Noticeably, an IC improvement can be observed from a visual point of view, which indicates that the detected targets are likely moving targets.

**Figure 27:**
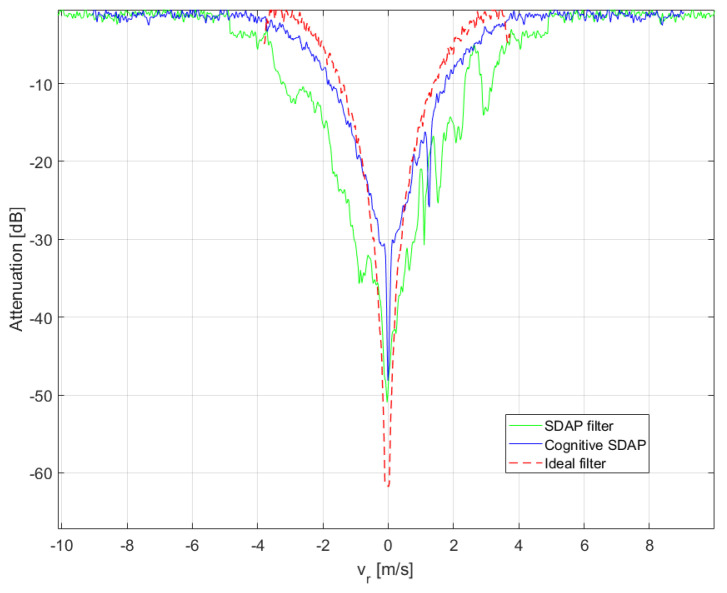
Filter comparison between cognitive SDAP filter and an ideal one.

The position (DN) and the bandwidth (DNB) of the SDAP filter notch, in the radial velocity domain, in then evaluated and compared with a reference Doppler profile. The reference Doppler profile is obtained by considering the minimum radial target velocity to be detected. More specifically, by assuming that that the minimum radial target velocity to detect is 2 m/s, then the reference Doppler profile can be obtained by using the minimum number of channels that are necessary to fullfil such requirement in ideal conditions (the term ideal condition indicates both homogeneous clutter and perfect estimation of the covariance matrix). A filter comparison is shown in Figure 27.

The red line represents the ideal filter whereas the blue trend shows the result relative to the C-SDAP filter. The DNB is defined as the ratio between the notch bandwidth of the Cognitive SDAP filter and the reference (ideal) one. In order to practically measure the DBN, we have considered the filter bandwidth measured at −10 dB. The closer the DNB ratio is to one, the more the SDAP performance approximates the ideal one. Therefore, the DNB is compared to a threshold to assess the SDAP performance, specifically $\lambda_{DNB}=1.5$. The notch bandwidth of the ideal filter corresponds to $DNB_{ideal}=2.8$ m/s whereas the notch bandwidth of the SDAP filter corresponds to $DNB_{SDAP}=3.48$ m/s. The measured DNB is equal to DNB=1.24, which satisfies the imposed condition. The DN, as already mentioned, represents the difference between the Doppler null of the cognitive SDAP filter and the ideal one. This is compared with a threshold that establishes the maximum tolerable difference. The tolerated difference between filters notches is set to $\lambda_{DN}=20$ dB. In our case, the measured DN is approximatively DN=13 dB. Furthermore, in this case, the corresponding threshold, imposed by $\alpha_{4}$, is not exceeded and, therefore, a spectrum analysis is not required, i.e., $\gamma_{3}=0$. It is worth pointing out that in Figure 27, the C-SDAP filter is also compared with the non-cognitive SDAP filter (black trend), where the training area is selected randomly (with no available a priori information). It is worth noting that the improvement obtained by using a cognitive approach is important.

## 6. Conclusions

Ground moving target imaging (GMTImg) has been studied by many researchers in the last decades. The research group at the University of Pisa and the Radar and Surveillance Systems National Laboratory has carried out important work that has been extensively reported in this feature paper. GMTImg is heavily based on the ability to suppress ground clutter and at the same time produce well-focussed images. As shown in this paper, a strong mathematical background has been laid to jointly remove clutter and produce ISAR images of moving targets. A novel approach based on Space-Doppler Adaptive Processing (SDAP) has been proposed that has laid the ground for the development of techniques that are applicable in practical scenarios. The theoretical findings have been well supported by simulation results and real data analysis. An important result has been achieved by introducing Virtual SDAP, namely V-SDAP, which makes GMTImg applicable also to single-channel radar systems, provided that a high PRF is sustainable. Results based on real data have shown that a virtual three channel SDAP outperforms a real two-channel SDAP. Last but not least, elements of cognition have been introduced to optimise the application of SDAP in heterogeneous and highly time and space-varying scenarios. Cognitive C-SDAP (SDAP) has demonstrated to outperform non-cognitive versions of SDAP algorithms.

## Figures and Tables

**Table 1:** Acquisition parameters. Left column: parameter definition, right column: parameter value.

Parameter	Value
Carrier frequency $f_{0}$	9.9 GHz
PRF	2.9 kHz
TX Bandwidth	600 MHz
ADC Sampling frequency	25 MHz
Platform Velocity	45 m/s
Incident Angle	$55^{\circ }$
Antenna Beamwidth	$\theta_{el}=20^{\circ }$, $\theta_{az}=20^{\circ }$
Acquisition Time	0.6 s
Platform Altitude	996 m
Baseline	0.08 m
Numbers of Rx channels	4

**Table 2:** Image Constrast before and after ISAR processing.

	${\mathrm{IC}}_{b}$	${\mathrm{IC}}_{a}$	$v_{r}$
Target 1	1.83	8.43	7.95 m/s
Target 2	2.91	9.86	3.75 m/s
Target 3	6.24	10.77	3 m/s

**Table 3:** Acquisition parameters. Left column: parameter definition, right column: parameter value.

Parameter	Value
Carrier frequency $f_{0}$	9.9 GHz
PRF	5 kHz
TX Bandwidth	120 MHz
ADC Sampling frequency	25 MHz
Platform Velocity	50 m/s
Incident Angle	$55^{\circ }$
Antenna Beamwidth	$\theta_{el}=20^{\circ }$, $\theta_{az}=7.5^{\circ }$
Acquisition Time	0.61 s
Platform Altitude	1200 m
Numbers of Rx channels	2

## Data Availability

The data comes from a private measurement campaign.

## List of Symbols

Additive noise	N(f,t)
Antenna azimuth aperture	$\theta_{az}$
Array dimension	$S_{array}$
Attenuation term	$J(y_{1})$
Average operation	$E\left\{\cdot \right\}$
Carrier frequency	$f_{0}$
Clutter signal return	$S_{c}\left(f,t\right)$
Clutter spatial correlation coefficient	$\rho_{s}$
Clutter spatial correlation coefficient	$\rho_{t}$
Convolution along time delay dimension	⊗
Convolution along Doppler frequency dimension	${⊗}_{m_{\nu }}$
Cross-range image size	$D_{y1}$
Cross-range resolution	$\delta_{az}$
Distance between radar platform and target reference point	$R_{0}\left(t\right)$
Doppler bandwidth	$B_{D}$
Doppler frequency	$f_{D}$
Effective rotation vector	$\Omega_{eff}$
Equivalent baseline	$b_{l,eq}$
Interference cross-power spectral matrix	$R_{Dc}$
Image contrast	IC
ISAR point spread function	I(τ,ν)
Multichannel range-Doppler image	$u_{D}$
Number of array channels	*P*
Number of range cells	$N_{r}$
Number of samples	$N_{samp}$
Numbers of unclassified pixels	$P_{NC}$
Numbers of classified pixels	$P_{C}$
Observation time	$T_{obs}$
Phase of the received signal	$\phi \left\{\cdot \right\}$
Platform Velocity	$v_{p}$
Pulse Repetition interval	$T_{R}$
Pulse Repetition Frequency	PRF

Radar wavelength	λ
Radial velocity	$v_{r}$
Range resolution	$\delta_{r}$
Received signal by the array element (p,q)	$S_{R}^{\left(p,q\right)}$
Received signal after motion compensation	$S_{R,C}$
Reference signal	$S_{ref}$
Reference vector	$\tilde{G}$
Rotation matrix	$M_{\xi x}$
Scatter position	x(t)
Segmentation controlling function	$\alpha_{1,b}$
Signal vector	$\tilde{S}$
Size of the illuminated swath along the range dimension	$D_{y2}$
Synthetic aperture	*L*
Speed of light in a vacuum	*c*
Signal bandwidth	*B*
Target reflectivity function	σ(y)
Target rotational motion velocity vector	$\Omega_{T}$
Target signal return	$S_{t}\left(f,t\right)$
Unit vector along the radar Line of Sight	$i_{LoS}\left(t\right)$
Vector of target-free training data	$\tilde{Z}$
Weight vector	$\tilde{W}$
Segmentation actuating function	$\gamma_{1}$
Training data selection controlling function	$\alpha_{2}$
Bayesian approach controlling function	$\alpha_{3}$
Training data selection actuating function	$\gamma_{2}$
Clutter suppression controlling function	$\alpha_{4}$
Clutter suppression actuating function	$\gamma_{3}$
Predefined clutter covariance matrix	$M_{c}$
Doppler Null	DN
Doppler Null Bandwidth	DNB
Frequency spectrum sensing	$F_{SS}$
